# Effects of clinical medications on male fertility and prospects for stem cell therapy

**DOI:** 10.3389/fcell.2023.1258574

**Published:** 2023-09-18

**Authors:** Peiting Mo, Ziran Zhao, Xinpeng Ke, Yong Fan, Chaohui Li

**Affiliations:** Key Laboratory for Major Obstetric Diseases of Guangdong Province, Department of Obstetrics and Gynecology, Key Laboratory of Reproduction and Genetics of Guangdong Higher Education Institutes, The Third Affiliated Hospital of Guangzhou Medical University, Guangzhou, Guangdong, China

**Keywords:** male fertility, clinical medications, spermatogenesis, stem cell therapy, exosomes therapy

## Abstract

An increasing number of men require long-term drug therapy for various diseases. However, the effects of long-term drug therapy on male fertility are often not well evaluated in clinical practice. Meanwhile, the development of stem cell therapy and exosomes treatment methods may provide a new sight on treating male infertility. This article reviews the influence and mechanism of small molecule medications on male fertility, as well as progress of stem cell and exosomes therapy for male infertility with the purpose on providing suggestions (recommendations) for evaluating the effect of drugs on male fertility (both positive and negative effect on male fertility) in clinical application and providing strategies for diagnosis and treatment of male infertility.

## 1 Introduction

In clinical practice, many reproductive-aged men require long-term drug therapy for various diseases, such as cardiovascular disease, tumor and chemoradiotherapy, hyperlipemia, depressive disorder and so on. However, the effects of drug therapy on male fertility are often not well evaluated. Drugs may affect male fertility by direct gonadal toxicity, altering the hypothalamic-pituitary-gonadal axis, causing sexual dysfunction, and negative effects on libido. Whereas, some small molecule drugs may have a positive protective effect on germ cell genesis and fertility maintenance during use. Thus, it is necessary to evaluate the reproductive effects before drugs approved for clinical use. Stem cell therapy and exosomes therapy are promising treatment options for male infertility. Mesenchymal stem cells (MSCs) and their exosomes have attracted much attention due to their anti-inflammatory and immunomodulatory properties, trophic properties, and anti-apoptotic properties, as well as their easy availability. This article mainly discussed the direct effect of drugs on male germ cell and its mechanism and summarized the therapeutic effect of stem cells and extracellular vesicles in this regard.

## 2 Discussion

Different small molecule medications have different pharmacological mechanisms and have varying effects on male fertility. Among the effects on male fertility, small molecule medications may affect male fertility by influencing testicular microenvironment, spermatogenesis, sperm capacitation, fertilization. The mechanism may not be singular. This article attempts to review the research on the mechanisms of action of small molecule medications at different stages of sperm development to fertilization, and to summarize the prospects and obstacles of stem cell therapy and exosomes in male fertility.

## 3 Conclusion

The influence of clinical drugs on male fertility is complex and needs comprehensive evaluation. Further researches are needed to better understand the impact of drugs on male fertility and provide evidence-based recommendations for clinical use. Although there are many problems that remains to be solved when using MSCs and exosomes in clinical practice, the continuous deepening of research and treatment methods may become emerging methods for treating male infertility and fertility preservation.

## 4 Main text

### 4.1 The effects of small molecule medications on male fertility

Fertility is defined as the capacity to establish clinical pregnancy. According to WHO, infertility refers to the inability to conceive after 12 months of regular unprotected sexual intercourse (Zegers-Hochschild et al., 2017). Between 8% and 12% of reproductive-aged couples are affected by infertility. 50% of couples have male factors in infertility (Vander and Wyns., 2018). The cause of male infertility can be divided into congenital, acquired, and idiopathic. The use of small molecule medications is one of the causes of male infertility (Agarwal et al., 2021). Long term drug treatment may damage spermatogenesis, change semen parameters, and lead to sexual dysfunction (Fode et al., 2021). The quantity and quality of sperm are important indicators for evaluating male fertility (Goossens and Tournaye., 2014), so reproductive history and semen analysis are necessary for the initial evaluation of male fertility. For males with initial evaluation abnormalities, further comprehensive evaluation by reproductive experts may be required (Agarwal et al., 2021). The main methods for treating male infertility include medication to improve sperm quality, application of assisted reproductive technology, treatment of diseases that lead to infertility, and the therapeutic effect of stem cells and their extracellular vesicles on male infertility (Agarwal et al., 2021).

#### 4.1.1 The effect of small molecule medications on testicular microenvironment

The main function of the scrotum-located testicles is to produce sperm and androgens. Spermatogenesis is a complex process of producing sperm, while the testicular microenvironment is crucial to the normal development of spermatogenesis (Horvath-Pereira et al., 2023). It regulates the proliferation, differentiation, and maturation of germ cell (Miyaso et al., 2022). The testicular microenvironment is composed of testicular cells and intercellular substance. Changes in immune homeostasis (Miyaso et al., 2022; Wang et al., 2018), somatic cells in testis (Zhao et al., 2020), blood–testis barrier, temperature, and the paracrine factors may all contribute to changes in the testicular microenvironment.

##### 4.1.1.1 Testicular weight

**FIGURE 1:**
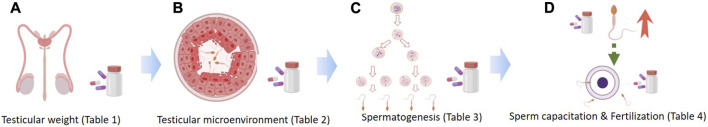
Effects and mechanisms of small molecule medications on male fertility. Small molecule medications effects on testicular weight **(A)**. testicular microenvironment **(B)**. spermatogenesis **(C)**. sperm capacitation and fertilization **(D)**. The targeted stages of medications, medicine categories, effects mechanisms, etc. are detailed in Tables 1–4.

During long-term drug therapy for various diseases, some medications may cause significant changes in testicular weight. The changes in testicular weight are also an indicator for evaluating the impact of drugs on male fertility (Figure 1A). In animal experiments, it has been found that fluoxetine, amlodipine, acetaminophen and nifedipine can decrease testicular weight (de Oliveira et al., 2013; Iranloye et al., 2009; Latif et al., 2008; Vieira et al., 2013; Wiger et al., 1995). Benzodiazepines as a sedative hypnotic drug have a negative impact on male fertility (Cook et al., 1979; Kar and Das., 1983; Means et al., 1982; Sanbuissho et al., 1995; Taher et al., 2019), but their impact on testicular weight is uncertain. Some experiments have shown that they do not cause changes in testicular weight (Chengelis et al., 1986), while others have shown that they can cause a decrease in testicular weight (Cook et al., 1979; Kar and Das., 1983; Means et al., 1982; Sanbuissho et al., 1995; Taher et al., 2019). Indometacin which used for pain relief and anti-inflammatory reduces or does not change testicular weight (Bagoji et al., 2017; Saksena et al., 1975). Statins which have lipid-lowering effect can improve testicular weight loss caused by hyperlipidemia (Shalaby et al., 2004), while Köhler-Samouilidis et al. found that Captopril which used to treat hypertension can increase testicular weight, although the study found that it has a negative impact on male fertility (Köhler-Samouilidis et al., 1997). The change of testicular weight is an indicator to evaluate the effect of drugs on male fertility in animal experiments, but it is not accurate, and needs to be evaluated together with other indicators. Table 1.

**TABLE 1:** The effects of small molecule medications on testicular weight.

Medicine categories		Relevant references	Species	Drug dosages	Durations
Benzodiazepines		Chengelis et al. (1986)	Dogs	75 mg/kg/day	10 days
		Chengelis et al. (1986)	Rats	550 mg/kg/day	28 days
		Cook et al. (1979)	Rats	50 mg/kg	10 days
		Kar and das (1983)	Mice	0.5 mg/day	15 days
		Means et al. (1982)	Dogs	30/100/300 mg/kg	2 weeks
		Sanbuissho et al. (1995)	Rats	0/20/40/80 mg/kg	2/4/9 weeks
		Taher et al. (2019)	Rats	2/5/10 mg/kg/day	8 weeks
SSRIs	fluoxetine	de Oliveira et al. (2013)	Rats	5/10/20 mg/kg	from day 13 gestation to day 21 lactation
CCB	amlodipine	Latif et al. (2008)	Rats	0.14 mg/kg	50 days
	Nifedipine	Iranloye et al. (2009)	Rats	0.57 mg/kg	30 days
NSAIDs	Acetaminophen	Wiger et al. (1995)	Mice	100–400 mg/kg	5 days

Abbreviation: SSRIs, selective serotonin reuptake inhibitors; CCB, calcium channel blocker; NSAIDs, Nonsteroidal Antiinflammatory Drugs.

##### 4.1.1.2 Testicular microenvironment

Apart from the testicular weight changes, testicular microenvironment also has a series of impact on spermatogenesis (Figure 1B). The use of common clinical drugs may lead to changes in the testicular microenvironment and thus affect spermatogenesis. In animal studies, it was found that bupropion (Yardimci et al., 2019), paroxetine (Erdemir et al., 2014; Yakubu and Atoyebi., 2018; Yardimci et al., 2019), benzodiazepines (Chengelis et al., 1986; Means et al., 1982), nifedipine (Iranloye et al., 2009; Lee et al., 2006), ethosuximide (Lee et al., 2006), valproate (Alsemeh et al., 2022; Nishimura et al., 2000; RØste et al., 2001; Sukhorum and Lamsaard., 2017), pregabalin (Shokry et al., 2020; Taha et al., 2020), rosuvastatin (Leite et al., 2017), etc., had negative effects on the testicular microenvironment. Antidepressant bupropion can cause testicular structural damage and interstitial edema (Yardimci et al., 2019). As a kind of selective serotonin reuptake inhibitors (SSRI), paroxetine can damage testicular structure, induce degeneration of seminiferous tubules, and cause vacuolization of germinal epithelium (Erdemir et al., 2014; Yakubu and Atoyebi, 2018; Yardimci et al., 2019). Meanwhile, there are differences in results among different studies on benzodiazepines. This may be related to the dosage used in animal experiments. When the dosage is 75 mg/kg body weight (Chengelis et al., 1986), it will cause spermatogenesis arrest and the germinal epithelium degeneration, but it will not cause abnormalities in Sertoli cells and Leydig cells. However, when the dosage is 100 mg/kg (Means et al., 1982), it will cause degeneration and necrosis of seminiferous tubules, and vacuolization of interstitial cells.

Calcium channel blockers which used for cardiovascular diseases are commonly believed to cause male infertility (Drobnis and Nangia, 2017). Voltage-gated Ca^2+^ (CaV) channels can be divided into different types of voltage-gated channel according to pharmacology. As antihypertensive drugs, Calcium channel blockers include L-type and T-type voltage-gated calcium channel inhibitor (Elmslie, 2004). It found that L-type and T-type voltage-gated calcium channel exist in male germ cell. However, the specific regulatory mechanism for male germ cell is not clear. In a study on the effect of nifedipine on male reproductive function in rats, after continuous administration of 0.57 mg/kg nifedipine for 30 days, no significant effects were observed on testicular histology (Iranloye et al., 2009). However, another study on male mice showed that high doses (100 mg/kg) of nifedipine or ethosuximide could cause immature development of seminiferous tubules, spermatogenic stagnated in the elongating spermatid stage, and poorly developed lumen. In the study, it was found that the mRNA expression of the transcription factor cAMP-responsive element modulator (CREM) activator isoform in the testes of experimental mice was increased together with the mRNA expression of transition protein 2 and protamine 2. It is suggested that the spermatogenic disorders caused by calcium channel blockers may be related to the ectopic expression of CREM-dependent genes in the testes (Lee et al., 2006). The difference in these two study results may also be related to different drug doses and durations of use. As an Antiepileptic drugs (AEDs), Valproic acid can lead to spermatogenic disorders (RØste et al., 2001), evidenced by degeneration of seminiferous tubules and loss and shedding of spermatogenic cells under microscopy (Nishimura et al., 2000). In addition, collagen deposition, widening of interstitial spaces, congestion of the tunica propria and interstitial ducts (Alsemeh et al., 2022), an increase in the percentage of spermatozoa with abnormal acrosomes (Sukhorum and Lamsaard., 2017), and the generation of multinucleated giant cells have been observed (Iamsaard et al., 2017). Studies have shown that valproic acid can induce tissue pathological damage associated with autophagy by modulating the AMPK/mTOR signaling pathway (Alsemeh et al., 2022). And another type of AEDs Pregabalin can reduce the number of germ cells while increasing the number of interstitial cells, and cause a decrease in the number and diameter of convoluted tubules (Salem et al., 2020). It also induces cell apoptosis, increases caspase-3 expression in the testis (Shokry et al., 2020), upregulates pro-apoptotic genes BAX and p38 MAPK, and downregulates the anti-apoptotic gene BCL2 (Taha et al., 2020). The reproductive toxicity of pregabalin may be related to the p38 MAPK and BAX/BCL2 signaling pathways (Taha et al., 2020).

##### 4.1.1.3 Oxidative stress

Oxidative stress is a cause of male infertility (Sharma et al., 2023). Increasing cellular oxidative stress is a pathway through which antiepileptic drugs damage male reproductive cells. Studies have shown that valproic acid, pregabalin, cannabidiol, and levetiracetam all increase oxidative stress in male reproductive cells (Baysal et al., 2017; Carvalho et al., 2022; Naderi et al., 2021; Ourique et al., 2016a; Ourique et al., 2016b; Shokry et al., 2020; Taha et al., 2020). Histologically, after administration of cannabidiol, the number of Sertoli cells will decrease (Carvalho et al., 2018), and testicular degeneration will occur (Patra and Wadsworth., 1991). The antidepressant drugs sertraline, fluoxetine, and citalopram also increase oxidative stress in the testes (Atli et al., 2017; Attia and Bakheet., 2013; Sakr et al., 2015). Taking fluoxetine during pregnancy and postpartum also has an impact on male reproductive health. Male offspring may exhibit reduced Sertoli cells in histology, decreased height of germinal epithelium, decreased diameter of seminiferous tubules, shorter epididymal tubules, and increased number of tubules without a lumen (de Oliveira et al., 2013; Ramos et al., 2015; Vieira et al., 2013). In animal experiments, it has been found that citalopram inhibits sperm production at different stages, leading to degeneration of seminiferous tubules, cell vacuolization, interstitial cell atrophy, and decreased sperm count in the lumen (Ilgin et al., 2017; Prasad et al., 2015). A 90-day animal study of long-term use of sertraline in rats showed that sertraline did not cause pathological changes in testicular tissue (Ghorbani et al., 2021). However, more animal studies have shown that long-term use of sertraline can cause histological damage to the testes (Atli et al., 2017; Hamdi, 2019). In animal studies, Antiviral drug such as lopinavir/ritonavir and nevirapine can increase oxidative stress in the testes (Adaramoye et al., 2012; 2015). Histologically, nevirapine causes extensive degeneration of the seminiferous tubules, necrosis and detachment of germ cells (Adaramoye et al., 2012). The animal study of Lopinavir/ritonavir found that the number of spermatogenic cells in the testis was reduced and the morphology was abnormal (Adaramoye et al., 2012; Adaramoye et al., 2015).

There are also some clinical drugs that can reduce testicular oxidative stress while treating diseases. For diabetic rats, antihypertensive drugs enalapril can reduce oxidative stress and downregulate the expression of NFκB and COX-2 (Kushwaha and Jena, 2012). Studies on experimental varicocele rats and rats with adjuvant arthritis have shown that celecoxib can reduce oxidative stress in testicular tissue and improve sperm quality (Darwish et al., 2014; Mazhari et al., 2018). In clinical studies of patients with hypercholesterolemia, statins have no effect on sperm quality (Bernini et al., 1998; Cai et al., 2008; Dobs et al., 2000; Purvis et al., 1992). In studies on rats fed a high-fat diet, statins have a protective effect on male reproductive function. They improve semen quality, increase serum testosterone levels, improve testicular weight loss caused by hyperlipidemia, and increase fertility index (Abdulwahab et al., 2021; Esmail et al., 2020; Shalaby et al., 2004). These reproductive protective effects may be achieved by enhancing the mTOR signaling pathway and reducing oxidative stress (Cui et al., 2017; Farsani et al., 2018; Gurel et al., 2019). However, studies on statins have found that Rosuvastatin has a negative impact on male fertility. This may be related to the lower age of its research subjects. Rosuvastatin is commonly used in children with dyslipidemia (Leite et al., 2017). Its research focuses on pre-pubertal. Studies have shown that rosuvastatin reduces sperm quality, increases oxidative stress, increases DNA damage, damages testicular tissue, causes pathological changes in testicular and epididymal tissue, reduces testosterone levels, and damages the distribution of steroid receptors (Leite et al., 2019; Leite et al., 2017; Leite et al., 2018). Additionally, research on antihypertensive drugs has shown that irbesartan and carvedilol play a protective role in testicular injury caused by diseases through anti-inflammatory, antioxidant and anti-apoptotic effects (Abu-Risha et al., 2022; Eid et al., 2016; Eid et al., 2019; Kabel et al., 2020; Ramzy et al., 2014). Table 2.

**TABLE 2:** The effects of small molecule medications on testicular microenvironment.

Medicine categories		Effects and mechanisms	Relevant references	Species	Drug dosages	Durations
SSRIs	bupropion	damage testicular structural and interstitial edema	Yardimci et al. (2019)	Rat	17 mg/kg	70 days
	paroxetine	damage testicular structure induce degeneration of seminiferous tubules vacuolization of germinal epithelium	Erdemir et al. (2014)	Rat	20 mg/kg	2 months
			Yakubu and Atoyebi, (2018)	Rat	10 mg/kg	21 days
			Yardimci et al. (2019)	Rat	3.6 mg/kg	70 days
	sertraline	increase oxidative stress	Atli et al. (2017)	Rat	5/10/20 mg/kg	28 days
		histological damage to the testes	Hamdi, 2019	Rat	15.63 mg/kg	28 days
	fluoxetine	sertoli cells↓(male offspring) height of germinal epithelium、diameter of seminiferous tubules↓(male offspring) shorter epididymal tubules (male offspring) number of tubules without a lumen↑(male offspring)	Sakr et al., 2015 de Oliveira et al., 2013	Rat Rat Rat Rat	10 mg/kg/day 5/10/20 mg/kg 7.5 mg/kg 7.5 mg/kg	28 days from day 13 gestation to day 21 lactation from the day 1 of pregnancy until 21 dab during pregnancy and lactation
			Ramos et al. (2015)			
			Vieira et al. (2013)			
	citalopram	sperm production↓sperm count in the lumen↓	Attia and Bakheet, (2013)	Mice Rat zebrafish	6/12/24 mg/kg/day	4/8 weeks
		degeneration of seminiferous tubules	Ilgin et al. (2017)		5/10/20 mg/kg/day	28 days
		cell vacuolization, interstitial cell atrophy	Prasad et al. (2015)		4/40/100 μg/L	2/4 weeks
benzodiazepines		arrest of spermatogenesis	Chengelis et al. (1986)	Dogs Rat Dog	75 mg/kg/day 550 mg/kg/day 30/100/300 mg/kg	10 days 28 days 2 weeks
		degeneration of the germinal epithelium, degeneration and necrosis of seminiferous tubules, vacuolization of interstitial cells	Means et al. (1982)			
CCB	Nifedipine	immature development of seminiferous tubules spermatogenic stagnated in the elongating spermatid stage poorly developed lumen	Iranloye et al. (2009)	Rat	0.57 mg/kg	30 days
			Lee et al. (2006)	Mice	1/10/100 mg/kg	7 days
	ethosuximide		Lee et al. (2006)	Mice	1/10/100 mg/kg	7 days
AEDs	valproic acid	the percentage of spermatozoa with abnormal acrosomes↑	Alsemeh et al. (2022)	Rat	100/300/500 mg/kg/day	8 days
		increase oxidative stress	Nishimura et al. (2000)	Rat	250/500/1000 mg/kg/day	4/7/10 weeks
		spermatogenic disorders, histological damage to the testes	RØste et al. (2001)	Rat	200/400 mg/kg bid	90 days
		generate multinucleated giant cells	Sukhorum and Iamsaard (2017)	Rat	500 mg kg/day	10 days
		modulate the AMPK/mTOR signaling pathway	Iamsaard et al. (2017)	Rat	500 mg/kg	10 days
	pregabalin	increase oxidative stress and cell apoptosis	Shokry et al. (2020)	Rat	300 mg/kg	60 days
		germ cells↓interstitial cells↑convoluted tubules↓	Taha et al. (2020)	Rat	300 mg/kg/day	90 days
		caspase-3 expression↑pro-apoptotic genes BAX and p38 MAPK↑the anti-apoptotic gene BCL2↓	Salem et al. (2020)	Rat	62 mg/kg/day	2 months
	cannabidiol	number of sertoli cells↓ testicular degeneration	Carvalho et al. (2018)	Mice Mice Mouse/human	15/30 mg/kg/day 50 mg/kg ∼100 μM	34 days 15/35 days 4/24 h
			Patra and Wadsworth (1991)			
			Li et al. (2022)			
	levetiracetam	increase oxidative stress	Baysal et al. (2017)	Rat	50/150/300 mg/kg	70 days
LLD	rosuvastatin	increase oxidative stress and DNA damage, sperm quality↓testosterone levels↓damage testicular tissue, distribution of steroid receptors	Leite et al. (2017)	Rat	3/10 mg/kg/day	30 days
PIR	lopinavir/ritonavir	spermatogenic cells↓increase oxidative stress, abnormal morphology spermatogenic cells	Adaramoye et al. (2015)	Rat	8.3/16.6 mg/kg	21 days
NNRTIs	nevirapine	increase oxidative stress, extensive degeneration of seminiferous tubules, necrosis detachment of germ cells	Adaramoye et al. (2012)	Rat	18/36 mg/kg	4 weeks
LLD	rosuvastatin	increase oxidative stress and DNA damage, sperm quality↓, testosterone levels↓	Leite et al. (2017)	Rat	3/10 mg/kg/day	30 days
		pathological changes in testicular and epididymal tissue	Leite et al. (2018)	Rat	3/10 mg/kg/day	49 days
		damages the distribution of steroid receptors	Leite et al. (2019)	Rat	3/10 mg/kg/day	30 days
LLD	statins	decrease oxidative stress the mTOR signaling pathway↑	Cui et al. (2017)	Rat	6 mg/kg/day	8 weeks
			Farsani et al. (2018)	Rat	20 mg/kg	8 weeks
			Gurel et al. (2019)	Rat	6 mg/kg	7 days
		improve semen quality and serum testosterone levels	Abdulwahab et al. (2021)	Rat	1 mg/kg	65 days
		improve testicular weight loss caused by hyperlipidemia	Esmail et al. (2020)	Rat	5 mg/kg/day	2 weeks
		improve fertility index	Shalaby et al. (2004)	Rat	1 mg/kg	65 days
AT2R	irbesartan	anti-inflammatory, antioxidant and anti-apoptotic	Abu-Risha et al. (2022)	Rat	100 mg/kg/day	15 days
NSBBs/α1receptor blocker	carvedilol	anti-inflammatory, antioxidant and anti-apoptotic	Eid et al. (2016)	Rat	10 mg/kg	2 weeks
			Eid et al. (2019)	Rat	10 mg/kg/day	20 days
			Kabel et al. (2020)	Rat	10 mg/kg/day	45 days
			Ramos et al. (2015)	Rat	1/10 mg/kg/day	4 weeks
ACEI	enalapril	decrease oxidative stress, the expression of NFκB and COX-2↓	Kushwaha and Jena. (2012)	Rat	10 mg/kg	4/8 weeks
NSAIDs	celecoxib	decrease oxidative stress	Darwish et al. (2014)	Rat	5 mg/kg	21 days
		sperm quality↑	Mazhari et al. (2018)	Rat	10 mg/kg	60 days

Abbreviation: SSRIs, selective serotonin reuptake inhibitors; CCB, calcium channel blocker; AEDs, Antiepileptic drugs; LLD, lipid-lowering drugs; PIR, protease inhibitors; NNRTIs, non-nucleoside reverse transcriptase inhibitors; AT2R, Angiotensin II, Receptor Blockers; NSBBs, non-selective beta-blockers; ACEI, Angiotensin-Converting Enzyme Inhibitors; NSAIDs, Nonsteroidal Antiinflammatory Drugs; dab:days after birth; bid, twice a day; ↓: decline; ↑: improve.

#### 4.1.2 The effect of small molecule medications on spermatogenesis

Spermatogenesis is through the proliferation of spermatogonia and differentiation to spermatocytes. After Meiosis, the spermatocytes that produce spermatozoa undergo round spermatids maturation, and finally form spermatozoa (Neto et al., 2016). Spermatogenesis is influenced by multiple factors, such as genetic disorders, environmental conditions, immune factors, etc. (Diemer and Desjardins, 1999; Chen et al., 2016; Gabrielsen and Tanrikut., 2016). For evaluating male fertility, semen analysis is an important indicator.

Small molecule medications can affect various germ cell during spermatogenesis (Figure 1C). The Angiotensin I-converting enzyme competitive inhibitor captopril reduces the proliferation rate of spermatogonial stem cells (Gao et al., 2018). Topiramate which used to patients with epilepsy can cause a decrease in the number of spermatogonia and spermatocytes (Otoom et al., 2004), resulting in the degeneration of spermatogenic cells and the formation of multinucleated giant cells (El et al., 2019), interstitial edema, and interstitial cell necrosis. Studies have found that topiramate downregulates the *VEGFA* gene, which promotes hormone entry into the vascular system in spermatogonia, and the *SYCP3* gene, which plays an important role in meiosis in spermatocytes. Cannabidiol which used to treat neuropsychiatric disorders can impair spermatogenesis, affect the mitosis and meiosis of germ cells (Patra and Wadsworth., 1991), inhibit the G1/S phase cell cycle transition, inhibit DNA synthesis, and downregulate key cell cycle proteins (Li et al., 2022).

Drugs can cause damage to male fertility by damaging DNA. Antiviral drug abacavir and etravirine (Matuszewska et al., 2021), lipid-lowering drugs rosuvastatin (Leite et al., 2017), paracetamol (Smarr et al., 2017) will increase DNA damage. Several studies have shown that various antidepressants have a damaging effect on sperm DNA. Some scholars have found that antidepressants promote DNA damage centered on telomeres in germ cells (Sołek et al., 2021). Paroxetine causes abnormal DNA fragmentation in sperm (Tanrikut et al., 2010). Sertraline and escitalopram increase DNA damage (Akasheh et al., 2014; Atli et al., 2017; Ilgin et al., 2017; Hamdi, 2019). *In vitro* studies of sertraline have found that it negatively affects fertilization by inhibiting specific calcium ion channels in sperm, which affect calcium influx (Rahban et al., 2021). In the molecular mechanism study of reproductive cell toxicity of antidepressants, scholars have found that the mechanism is mediated by oxidative-reductive balance disorder, enzyme and non-enzyme cell protection mechanism failure, and mitochondrial dysfunction. Antidepressants can cause defects in spindle assembly and improper organelle segregation during *in vitro* cell division (Solek et al., 2021). For diabetic rats, enalapril can reduce sperm DNA damage caused by the disease (Kushwaha and Jena., 2012).

Small molecule drugs can also affect sperm quality by regulating gene expression. In research on antiepileptic drugs, it has been shown that they have a negative impact on sperm quality, but the mechanisms involved may vary. With the regard to carbamazepine, research has found that it downregulates the expression of potassium voltage-gated channel subfamily J member 11 (KCNJ11) in the testis, upregulates microRNA let-7a expression, and is statistically correlated with decreased sperm motility and increased sperm tail defects. Conversely, it upregulates cystic fibrosis transmembrane conductance regulator (CFTR) and microRNA 27a expression and is positively correlated with sperm tail defects (Tektemur et al., 2021). Previous studies have shown that decreased CFTR expression in sperm is associated with decreased sperm quality (Li et al., 2010). The authors speculate that the results contradicting previous research may be due to CFTR mRNA upregulation caused by CFTR channel dysfunction. The expression of CFTR and microRNA is related to sperm quality, but further experimental research is needed to confirm whether carbamazepine causes sperm damage through KCNJ11 or CFTR channels. Regarding valproic acid, research has found that the decreased expression of phosphorylated protein and Ki67 in the testis may affect the formation of the acrosome during sperm generation, leading to premature acrosome reactions and abnormal sperm heads. In clinical studies, the novel AED oxcarbazepine has been shown to increase sperm motility, concentration, and vitality (Guo et al., 2021; Wu et al., 2018). The authors speculate that this may have a positive effect on sperm production and maturation by raising FSH and LH levels. In addition to affecting sperm quality, small molecule drugs may also affect male fertility by affecting testosterone levels. Beta receptor antagonists such as propranolol and atenolol, also known as beta blockers, have been shown to reduce sperm motility, increase sperm abnormality rate, and lower testosterone levels, but they do not cause changes in the histology of the reproductive organs (El-Sayed et al., 1998; Fogari et al., 2002; Rosen et al., 1988; Suzuki et al., 1988). Studies on atenolol suggest that the reduction in testosterone levels may be due to the inhibition of testosterone release by reducing cAMP production in interstitial cells, rather than affecting steroidogenic enzyme activity (Khan et al., 2004). *In vitro* studies have shown that atenolol can increase sperm motility, and this increase in movement rate is downregulated by calcium channel blockers. Researchers speculate that this may be due to the fact that sperm adrenaline regulation may be calcium-dependent (Nelson and Cariello., 1989). Studies on propranolol have shown that it has a negative effect on male reproductive function. In the study on the mechanism of Propranolol inhibiting sperm motility, it was found that the isomer D-propranolol, which does not have beta receptor blocking activity, has a significant inhibitory effect on human sperm motility. Researchers speculate that propranolol’s inhibitory effect on sperm motility is due to its local anesthetic properties rather than beta receptor blockade (White et al., 1995). Other studies on propranolol have shown that it reduces sperm motility, increases sperm abnormality rate, lowers sperm concentration, reduces sperm count, inhibits sperm capacitation, acrosome reaction, and fertilization (De Turner et al., 1978; Khaled et al., 2020; Nusier et al., 2007; White et al., 1995). However, it does not cause chromosomal aberrations in germ cells (Aruna and Krishnamurthy, 1986). Table 3.

**TABLE 3:** The effects of small molecule medications on spermatogenesis.

Medicine categories		Effects	Mechanisms	Relevant references	Species	Drug dosages	Durations
ACEI	captopril	affect various germ cell during spermatogenesis	the proliferation rate of spermatogonial stem cells↓	Gao et al. (2018)	Mice	0.001 M/0.01 M	∼4 days
AEDs	topiramate		spermatogonia and spermatocytes↓histological damage to the testes	Otoom et al. (2004)	Rat	100 mg/kg	60 days
			downregulate the VEGFA gene and the SYCP3 gene	El et al. (2019)	Mice	100/200/400 mg/kg	4 weeks
	cannabidiol		impair spermatogenesis, affect the mitosis and meiosis of germ cells	Patra and WAdsworth (1991)	Mice	50 mg/kg	15/35 days
			inhibit the G1/S phase cell cycle transition and DNA synthesis	Li et al. (2022)	Mice/human	∼100 μM	4/24 h
NRTIs	abacavir	cause damage to male fertility by damaging DNA	increase DNA damage	Matuszewska et al. (2021)	Rat	60 mg/kg	16 weeks
NNRTIs	etravirine			Matuszewska et al. (2021)	Rat	40 mg/kg	16 weeks
LLD	rosuvastatin			Leite et al. (2017)	Rat	3/10 mg/kg/day	30 days
NSAIDs	paracetamol			Smarr et al. (2017)	Human	not mentioned	not mentioned
SSRIs	paroxetine		abnormal DNA fragmentation in sperm	Tanrikut et al. (2010)	Human	not mentioned	5 weeks
	sertraline		increase DNA damage	Akasheh et al. (2014)	Human	25/50 mg/days	1 weeks/3 months
				Atli et al. (2017)	Rat	5/10/20 mg/kg	28 days
				Hamdi (2019)	Rat	15.63 mg/kg	28 days
	escitalopram			Ilgin et al. (2017)	Rat	5/10/20 mg/kg	28 days
ACEI	enalapril		reduce sperm DNA damage caused by the disease	Kushwaha and Jena (2012)	Rat	10 mg/kg	4/8 weeks
AEDs	carbamazepine	affect sperm quality by regulating gene expression	the expression of KCNJ11 in the testes↓microRNA let-7a, CFTR, and microRNA 27a↑	Tektemur et al. (2021)	Rat	25 mg/kg/day	60 days
	valproic acid		negative effects of acrosome formation during spermatogenesis (expression of phosphorylated proteins and Ki67↓) premature acrosome reactions and abnormal sperm heads	Alsemeh et al. (2022)	Rat	100/300/500 mg/kg/day	8 days
				Nishimura et al. (2000)	Rat	250/500/1000 mg/kg/day	4/7/10 weeks
				RØste et al. (2001)	Rat	200/400 mg/kg bid	90 days
				Sukhorum and Iamsaard (2017)	Rat	500 mg/kg	10 days
	oxcarbazepine		positive effect on sperm production and maturation by raising FSH and LH levels	Guo et al. (2021)	Human	706 ± 182 mg/day	≥6 months
				Wu et al. (2018)	Human	300–900 mg/day	≥6 months
β-receptor antagonists		affect testosterone levels	reduce sperm motility increase sperm abnormality rate lower testosterone levels	El-Sayed et al. (1998)	Rat	atenolol:9/18 mg/kg	60 days
				El-Sayed et al. (1998)	Rat	metoprolol:3.5/7 mg/kg	60 days
				El-Sayed et al. (1998)	Rat	propranolol:7.5/15 mg/kg	60 days
				Fogari et al. (2002)	Human	valsartan:80 mg/day	16 weeks
				Fogari et al. (2002)	Human	atenolol:50 mg/day	16 weeks
				Rosen et al. (1988)	Human	propranolol:80 mg bid	1 week
				Rosen et al. (1988)	Human	metoprolol:100 mg bid	1 week
				Rosen et al. (1988)	Human	atenolol:100 mg/day	1 week
				Rosen et al. (1988)	Human	pindolol:10 mg bid	1 week
				Suzuki et al. (1988)	Human	atenolol:50–100 mg	1 years

Abbreviation: ACEI, Angiotensin-Converting Enzyme Inhibitors; AEDs, Antiepileptic drugs; NRTIs, nucleoside reverse transcriptase inhibitors; NNRTIs, non-nucleoside reverse transcriptase inhibitors; LLD:lipid-lowering drugs; NSAIDs, Nonsteroidal Antiinflammatory Drugs; SSRIs, selective serotonin reuptake inhibitors; bid:twice a day; ↓: decline; ↑: improve.

#### 4.1.3 The effect of small molecule drugs on sperm capacitation and fertilization

Spermatozoa passing through female reproductive tract fertilizes egg and provides its genetic material. Before fertilization, spermatozoa require capacitation which is a process that sperm acquires the capacity to fertilize. This capacity includes hyperactivated movement, the acrosome reaction and fusion with oocytes (Bailey, 2010). The fertilization process is influenced by factors such as sperm motility and female reproductive tract environment (Fraser, 1992).

Male gametes must undergo the process of capacitation to meet the oocyte prior to fertilization (Figure 1D). Propranolol have shown a negative effect on male reproductive function as it inhibits sperm capacitation, acrosome reaction, and fertilization (White et al., 1995). Animal experiments with ibuprofen have shown that it weaken the ability of sperm to fertilize *in vitro* and reduces adult fertility potential (Barbosa et al., 2020; Martini et al., 2008). *In vitro* studies of sertraline have found that it negatively affects fertilization by inhibiting specific calcium ion channels in sperm, which affect calcium influx (Rahban et al., 2021). Animal experiments have shown that lisinopril reduces zona pellucida penetration and acrosome reaction (Saha et al., 2000a). However, there are also animal experiments and clinical studies that suggest a positive effect of lisinopril on male reproductive function. It can increase the number and quality of sperm and improve fertility (Mbah et al., 2012; Okeahialam et al., 2006). Calcium ions are important ions that trigger the acrosome reaction (Hong et al., 1984). Nimodipine and verapamil can cause a decrease in sperm density and motility and a decrease in acrosome reaction (Juneja et al., 1990; Saha et al., 2000b). It was found that captopril inhibits the acrosome reaction and reduces the proportion of penetrating oocytes (Foresta et al., 1991).

There are also drugs that have a positive effect on sperm capacitation and fertilization (Figure 1E). Many clinical studies have shown that indomethacin can improve sperm production in patients with oligo asthenoter atozoospermia, increase sperm motility and sperm count, and enhance fertilization ability. However, it can decrease testosterone levels (Aydin et al., 1995; Barkay et al., 1984; Conte et al., 1985; Guo et al., 2015). In studies on the effect of sodium ions on sperm motility, amiloride was found to increase the acrosome reaction and decrease sodium-induced sperm motility (Peris et al., 2000; Rai and Nirmal., 2003; Wong et al., 1981). Table 4.

**TABLE 4:** The effects of small molecule medications on sperm capacitation and fertilization.

Medicine categories		Effects and mechanisms	Relevant references	Species	Drug dosages	Durations
β-blocker	propranolol	inhibit sperm capacitation、acrosome reaction and fertilization	White et al. (1995)	Human	5/50/500 μM	0–240 min
NSAIDs	ibuprofen	weak sperm fertilization ability *in vitro*	Barbosa et al. (2020)	Rat	0/2.4/7.2/14.3 mg/kg/day	30 days
			Martini et al. (2008)	Mice	5.6/11.2/16.8 mg/kg/day	35/60 days
SSRIs	sertraline	negatively affects fertilization by inhibiting specific calcium ion channels	Rahban et al. (2021)	Human	10/20 μM	∼60 min
CCB	nimodipine	decrease in sperm density and motility	Saha & Bhargava et al., 2000	Rat	40 mg/60 mg/kg/day	2/6 weeks
	verapamil	inhibits the acrosome reaction	Juneja et al. (1990)	Pigs	1/5/10 mg/kg	4/12 weeks
ACEI	lisinopril	reduce zona pellucida penetration and acrosome reaction	Saha & Garg et al., 2000	Rat	10/20 mg/kg/day	2/6 weeks
	captopril	inhibit the acrosome reaction	Foresta et al. (1991)	Human	10/50/100 nmol/L	0–18 h
NSAIDs	indomethacin	improve sperm production in patients with oligoasthenozoospermia increase sperm motility and sperm count enhance fertilization ability	Aydin et al. (1995)	Human	75 mg/day	12 weeks
			Barkay et al. (1984)	Human	25 mg bid/tid/qid; 50 mg tid	60 days
			Conte et al. (1985)	Human	100 mg	30 days
			Guo et al. (2015)	Human	25 mg bid	3 months
diuretic	amiloride	increase the acrosome reaction decrease sodium-induced sperm motility	Peris et al. (2000)	Ram	10^–7^∼10^−4^M	30 min
			Rai and Nirmal (2003)	Lizard	1–100 μM	1 h
			Wong et al. (1981)	Rat	10^–5^∼3*10^−3^M	3 h

Abbreviation: NSAIDs, Nonsteroidal Antiinflammatory Drugs; SSRIs, selective serotonin reuptake inhibitors; CCB, calcium channel blocker; ACEI, Angiotensin-Converting Enzyme Inhibitors; bid, twice a day; tid, three times a day; qid, four times a day.

### 4.2 Stem cell therapy in male infertility

**FIGURE 2:**
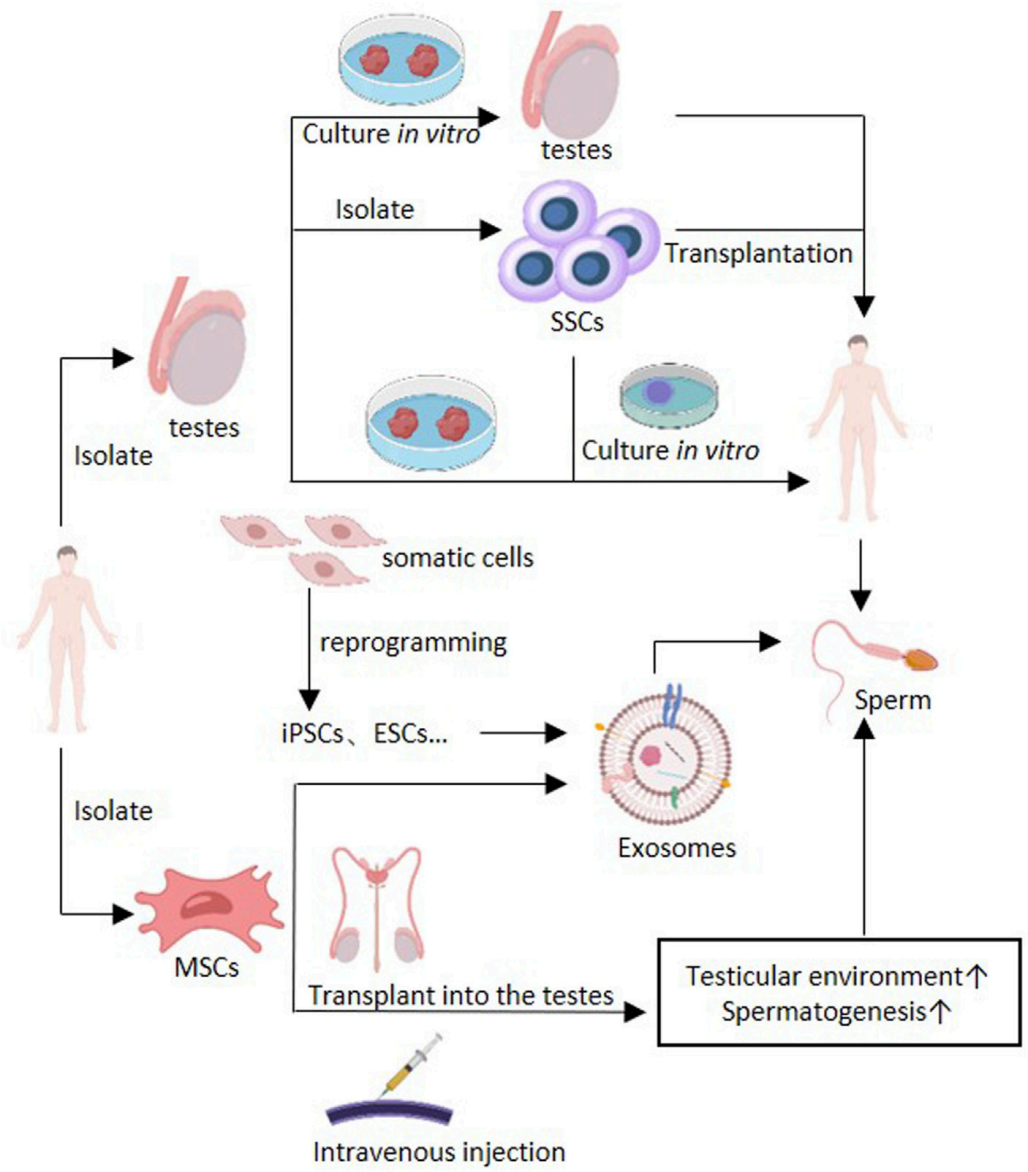
Application of stem cell therapy in male infertility. The separated MSCs can be divided into BM-MSCs, ADSC, AFSCs, USCs, UMSCs, etc., for therapy use. MSCs can improve testicular microenvironment and spermatogenesis by intravenous injection or testicular transplantation. Exosomes isolated from mesenchymal stem cells also have the same effect. Exosomes can also be isolated from ESCs and iPSCs, etc. Treatment may also be achieved through autologous transplantation of SSCs, autologous transplantation of testicular tissue and *in vitro* induction of spermatogenesis (culture testicular tissue or SSCs *in vitro* culture system to produce sperm). Abbreviations: Bone marrow derived MSCs (BM-MSCs), adipose derived stem cells (ADSCs), amniotic fluid-derived stem cells (AFSCs), Urine-derived stem cells (USCs), umbilical mesenchymal stem cells (UMSCs), embryonic stem cells (ESCs), induced pluripotent stem cells (iPSCs), spermatogonial stem cells (SSCs), ↑: improve.

With the rapid development of stem cell therapy these years, its application in male infertility has also received many attentions. Stem cells have the ability to self-renew and can differentiate into other special cell types (Ismail et al., 2023). In the treatment of male infertility, stem cells mainly play a therapeutic role through two pathways. One is to improve the microenvironment of spermatogenesis to restore the function of the remaining spermatogonial stem cells (SSCs). The other is to form germ cells through stem cells and further form sperm. At present, research on stem cell therapy mainly focuses on the application of MSCs (Ganjibakhsh et al., 2022; Ismail et al., 2023; Qiu et al., 2023). In addition, the vesicle system produced by stem cells, such as exosomes, also plays an important role in the field of stem cell therapy (Balistreri et al., 2020). The treatment methods of MSCs and exosomes in male infertility are illustrated in Figure 2.

#### 4.2.1 MSCs in male infertility

MSCs are adult stem cells that can be isolated from many tissue such as adipose tissue, bone marrow or cord blood.It is still unclear whether the therapeutic effect of MSCs on male infertility is achieved by improving the testicular microenvironment or by differentiating into germ cell (Badawy et al., 2020). MSCs have the advantages of relatively easy access and low transplant rejection reaction. Many scholars have attempted to differentiate MSCs into male germ-like cells (Behzadi et al., 2019; Luo et al., 2019) and even haploid specialized like cells (Shlush et al., 2017). However, it has not yet been achieved to differentiate sperm from MSCs and produce offspring. Further investigation is still needed in this regard (Zhang et al., 2014). The different sources of MSCs can be divided into bone marrow derived MSCs (BM-MSCs), adipose derived stem cells (ADSCs), amniotic fluid-derived stem cells (AFSCs), Urine-derived stem cells (USCs), umbilical mesenchymal stem cells (UMSCs), etc., The research content mainly focuses on the improvement of MSCs on the damaged testicular microenvironment and spermatogenesis, and on the differentiation of MSCs into germ cell. In the research, the cells that have received much attention include BM-MSCs and UMSCs (Fazeli et al., 2018). However, obtaining BM-MSCs is more difficult compared to other types of MSCs. USCs and their exosomes isolated from the urine of healthy men have been found to promote the recovery of spermatogenesis in azoospermic mice (Deng et al., 2019). This non-invasive and simpler method of obtaining MSCs has advantages in widespread clinical applications.

In the study of mice, Co-transplantation of MSCs improves SSCs transplantation efficiency (Kadam et al., 2018). Transplantation of BM-MSCs alone can upregulate the expression of target genes related to spermatogenesis and improve fertility of azoospermic rats (Badawy et al., 2020) *In vitro* studies, it can also better maintain spermatogenesis in co-culture with SSCs (Önen et al., 2023). For the administration method, intravenous injection of MSCs can alleviate the testicular toxicity caused by gonadal toxic substances through antioxidant, anti-inflammatory, and anti-apoptotic effects (Abdelaziz et al., 2019; Elbaghdady et al., 2018; Sherif et al., 2018), and testicular injection also has an improvement effect (Hsiao et al., 2015; SM et al., 2017). ADSCs and AFSCs can reduce oxidative stress in the testes and promote sperm production, (Eliyasi et al., 2020; Ibrahim et al., 2021; Qian et al., 2020; Siregar et al., 2021), and can improve the damage of chemotherapy drugs to testes (Meligy et al., 2019). Intratesticular injection of ADSCs can improve the spermatogenesis of azoospermia (Ganjibakhsh et al., 2022; Hajihoseini et al., 2018; Karimaghai et al., 2018), while xenotransplantation of human umbilical cord blood derived MSCs into azoospermia mice also has the same effect (Abd et al., 2017). Animal experiments have shown that after transplantation of BM-MSCs into the testis, in addition to influencing male reproductive function through paracrine action, they will also relocate to a new niche to form colonies, thus realizing the reconstruction of the damaged testicular germinal epithelium (Ghasemzadeh-Hasankolaei et al., 2016; Monsefi et al., 2013). Scholars try to differentiate different types of MSCs into germ cell, hoping that they can become a source of germ cell for the treatment of male infertility (Dissanayake et al., 2018; Ghaem et al., 2018; Kumar et al., 2018; Liu et al., 2018; Shlush et al., 2017). However, this goal has not yet been achieved. Table 5.

**TABLE 5:** Clinical trials on stem cell therapy for male infertility (U. S. National Library of Medicine).

Nct number	Stem cell type	Sex	Phases	Study status	Study results
NCT02025270	BM-MSCs	MALE	PHASE1|PHASE2	COMPLETED	NO
NCT02641769	BM-MSCs	MALE	PHASE1|PHASE2	UNKNOWN	NO
NCT02041910	BM-MSCs	MALE	PHASE1|PHASE2	UNKNOWN	NO
NCT03762967	ADSCs/SVF	MALE	PHASE2	UNKNOWN	NO
NCT02008799	BM-MSCs	MALE	NA	UNKNOWN	NO
NCT05158114	UC-MSCs	ALL	PHASE1	RECRUITING	NO
NCT02414295	MSCs	MALE	NA	COMPLETED	NO
NCT04452305	SSCs	MALE	NA	RECRUITING	NO

Abbreviation: NA, Not Applicable is used to describe trials without FDA-defined phases, including trials of devices or behavioral interventions. BM-MSCs, bone marrow derived MSCs; ADSCs, adipose derived stem cells; SVF, Stromal Vascular Fraction; UC-MSCs:umbilical cord derived mesenchymal stem cells; MSCs, mesenchymal stem cells; SSCs, spermatogonial stem cells.

In addition to tissue-originated MSCs, pluripotent stem cells MSCs (PSC-MSCs) are another pathway to obtain MSCs. PSC-MSCs have attracted attention due to their easier availability, more convenience quality control, and possibly large-scale production compared to tissue-originated MSCs (Abdal et al., 2019; Sabapathy and Kumar., 2016). In the research of PSC-MSCs in the treatment of diseases, PSC-MSCs having anti-inflammatory and immunomodulatory properties, like tissue-originated MSCs, can play a therapeutic role via paracrine, exosomes (Qu et al., 2022). However, it has the same safety issue of tumorigenicity as tissue-originated MSCs and iPSCs (Jiang et al., 2019). Although PSC-MSCs have been studied to varying degrees in the treatment of other diseases, there is still a lack of research in the treatment of male infertility.

MSCs have anti-inflammatory and immunomodulatory properties, trophic properties, and anti-apoptotic properties (Murphy et al., 2013). It may improve male infertility in multiple ways. More research is needed on the mechanism of its action. From the perspective of clinical treatment, different types of MSCs have their own advantages, and more research and analysis are needed to determine which type of MSCs treatment is more effective and in what manner (Zhankina et al., 2021). The safety of various mesenchymal cell applications also needs further exploration (Pendleton et al., 2013).

#### 4.2.2 iPSC/ESC in male infertility

In addition, embryonic stem cells (ESCs) and human induced pluripotent stem cells (iPSCs) have also received attention in this field. Human ESCs and iPSCs are two pluripotent populations that can self-renew and differentiate into any type of cell (Zhao et al., 2021). iPSCs were discovered in 2006 by Takahashi and Yamanaka as pluripotent stem cells that exhibit the morphological and growth characteristics of ESCs and express ESCs marker genes (Takahashi and Yamanka., 2006). The male germ cell differentiated from iPSCs is a germ cell with the patient’s own genetic material, which is the advantage of iPSCs as a cell therapy. However, iPSCs also reduces the immune system response of patients after transplantation. The disadvantage of iPSCs lies in the possibility of tumor formation, and its safety still needs to be considered (Sojoudi et al., 2023). Although it has been found in research that human iPSCs can directly differentiate into haploid spermatogenic cells, it is still not possible to ultimately form personalized human gametes *in vitro* (Easley et al., 2012; Eguizabal et al., 2011). Improving the efficiency of differentiation, inducing iPSC to produce personalized human gametes and identify markers for successful differentiation of germ cell are the difficulties that need to be broken through (Zhao et al., 2021). Although in the research of mice, embryonic stem cells can complete Meiosis *in vitro* and produce offspring through assisted reproduction technology (Nayernia et al., 2006; Zhou et al., 2016). However, this technology has not yet been achieved in the study of human ESCs. And there are differences in the genetic material between the germ cell obtained from ESCs and the recipient, which will lead to ethical disputes (Meligy et al., 2019).

#### 4.2.3 SSCs in male infertility

The use of patients’ own germ cell for treatment can mainly be achieved through autologous transplantation of SSCs, autologous transplantation of testicular tissue and *in vitro* induction of spermatogenesis (Figure 2), but these approaches have not yet been applied to clinical practice, are still in the experimental stage, and there are many problems to be solved (Dong et al., 2019). SSCs can maintain self-renewal and differentiate into sperm, providing genetic material for the next-generation. SSCs can differentiate into spermatozoa *in vitro* and generate mature spermatozoa through Spermatogenesis by transplantation *in vivo*, thus restoring male fertility (Diao et al., 2022). However, there is still no systematic method for long-term cultivation of human SSCs *in vitro*. Whether the epigenetic stability can be maintained in long-term *in vitro* culture still needs further exploration, although some studies have found that the sperm DNA methylation of the offspring of long-term *in vitro* cultured mouse SSCs after transplantation is stable (Serrano et al., 2023), and the genetic and epigenetics stability of human spermatogonial stem cells in long-term culture (Nickkholgh et al., 2014). SSCs transplantation was first achieved in mice in 1994 (Brinster and Zimmermann, 1994). In recent years, Whelan et al. have found that transplanting rat spermatogonial stem cells that have been frozen for over 20 years into mice can still produce sperm (Whelan et al., 2022). However, there is still limited research on spermatogonial stem cell transplantation in humans. Up to now, it has not been possible for human SSCs to form gametes *in vitro*, nor for cryopreserved human testicular tissue to initiate spermatogenesis *in vitro* (Portela et al., 2019). Autologous testicular tissue transplantation may be an option for men who want to receive gonadal toxicity treatment before puberty (Duca et al., 2019). Re transplantation of frozen testicular tissue into patients can achieve spermatogenesis *in vivo*, thus restoring the fertility of patients. In the study of primates, scholars have restored male fertility and produced offspring through cryopreserved autologous testicular tissue transplantation (Fayomi et al., 2019; Jahnukainen et al., 2012). Although many centers have also cryopreserved testicular tissue from pre-adolescent patients, testicular tissue transplantation has not yet been achieved in humans (Goossens et al., 2020; Valli-Pulaski et al., 2019; Zarandi et al., 2018). And for patients with cancer, there may be a risk of cancer cells in the tissue (Abdelaal et al., 2021; Sanou et al., 2022). Transplantation may lead to tumor recurrence in the recipient (Hou et al., 2007; Jahnukainen et al., 2001). Therefore, the safety of frozen testicular tissue autotransplantation for tumor patients should be comprehensively evaluated. For prepubertal patients, the testicular tissue is relatively small and the number of spermatogonial stem cells is limited. Therefore, expanding the number of spermatogonial stem cells *in vitro* before transplantation is also a challenge that needs to be overcame (Zarandi et al., 2018). In addition, some studies have shown that the germ cell in the male testicular tissue before puberty may have abnormal maturation of germ cell, which may lead to the failure to restore fertility after reimplantation of testicular tissue (Pietzak et al., 2015). Further exploration is needed on the conditions for long-term stable cryopreservation of testicular tissue and the location of transplantation (Moraveji et al., 2019; Sharma et al., 2019). The above technologies have not yet reached the clinical stage (Diao et al., 2022; Martin-Inaraja et al., 2021; Sanou et al., 2022).

Different stem cells have their own advantages and disadvantages when applied to male infertility. MSCs and SSCs are the existing stem cell options for male infertility. According to the data of U. S. National Library of Medicine, there are 8 clinical trials on stem cell therapy for male infertility (Table 5), but none of them has submitted any results yet. No relevant clinical trial results were found in PubMed. Further exploration of its mechanism of action and individualized selection of stem cell therapy regimens may be necessary.

Consequently, MSCs and SSCs may have more applications in the treatment of male infertility compared to other types of stem cell. The reason why MSCs have attracted much attention from researchers is that they are easy to obtain and cultivate and can achieve therapeutic effects through autologous cell transplantation. Different types of MSCs have different advantages. Choosing which MSCs to use in treatment may require more research and individualized treatment programs. Forming sperm from stem cells may be a potential therapeutic treatment for male infertility, although it has not yet been achieved. SSCs play an important role for male fertility preservation, although more research is needed in areas such as cryopreservation and *in vitro* culture. The application of stem cells in the treatment of male infertility still requires more extensive research.

### 4.3 The application of exosomes in male infertility

Exosomes are extracellular vesicles that are released from most cell types. Exosome play an important role in intercellular connections. Its diameter is 60–180 nm, which can be considered as an extracellular organelle mainly composed of proteins, lipids, and nucleic acids that act through endocytosis, phagocytosis, and membrane fusion (Sojoudi et al., 2023; Wang, 2021). Stem cell exosomes are another important pathway for stem cell therapy. A cell free therapy can be achieved by obtaining stem cell exosomes (Balistreri et al., 2020). In recent years, the function of exosomes in male reproduction has received widespread attention. The miRNA expression of extracellar microvessels in oligozoospermia and necropermia patients is different from that in healthy men (Abu-Halima et al., 2016). Other studies have shown that harmful substances can cause damage to sperm development by damaging exosomal secretion associated with spermatozoa (Ali et al., 2023). In terms of treatment, exosomes have advantages such as low immunogenicity, easy management, and no risk of tumor development, and can be used as a cell-free therapeutic approach (Taheri et al., 2019). Exosomes may be applied as novel drugs in the clinical treatment of male infertility.

Various types of cells in the testes secrete extracellular vesicles, with the extracellular vesicles secreted by the epididymis and prostate being the most concerned. The signal pathway proteins and miRNAs related to spermatogenesis in these exosomes may become a new therapeutic method for male reproduction and a means of disease diagnosis (Amiri et al., 2023; Candenas and Chianese, 2020; Eikmans et al., 2020; Lal et al., 2022; Zhu et al., 2020). Semen extracellular vesicles from the epididymis and prostate are known as epididymosomes and prostasomes, respectively. Sperm motility activation, capacitation and acrosome reaction are related to epididymosomes and prostasomes (Simon et al., 2018). In current research, it has been found that epididymosomes are involved in sperm maturation and have the function of reducing oxidative stress, regulating gene expression in sperm, regulating sperm morphology and motility. Prostasomes play an important role in regulating sperm motility and sperm capacitation (Baskaran et al., 2020; Ma et al., 2023). In addition, exosomes that sertoli cells secretion also play an important role in male fertility, although the small molecules it plays are still not very clear (Gao et al., 2023) Sertoli-derived exosomes can reduce oxidative stress and improve the microenvironment of spermatogenesis (Salek et al., 2021). Some studies on pigs have shown that the exosomes secreted by Sertoli cells can promote the proliferation of spermatogonial cells. This means that exosomes may be used for feeder-free layer culture systems (Thiageswaran et al., 2022). Studies have found that extracellular vesicles in semen also play an important role in embryonic development before implantation (Chen et al., 2020). In addition, exosomes derived from MSCs also have good prospects in the treatment of male infertility.

Research has shown that the exosomes of MSCs from different sources have an improvement effect on testicular injury (Mobarak et al., 2021; Sagaradze et al., 2019; Zhang et al., 2018b), which may be due to anti-inflammatory, antioxidant, and anti-apoptotic effects (Zhang et al., 2018a) (Table 6). In general, exosomes can play an anti-inflammatory, anti-hypoxic, anti-apoptotic and other roles by transmitting miRNA and other substances, improve the testicular microenvironment, and thus improve spermatogenesis, fertilization, implantation, and other aspects. However, no clinical trial was found in the U. S. National Library of Medicine or PubMed for the treatment of testicular injury with exosomes currently. There are animal studies on the treatment of testicular injury with exosomes. Radiation-induced testicular injury can be alleviated by macrophage-derived exosomes which contain protective components G-CSF and MIP-2 (Liu et al., 2020). The advantage of exosome is to avoid the degradation of active ingredient and the disadvantage of cell therapy. It is easy to detect the exosomes released by testicular cells in seminal plasma. With the deepening of research on its mechanism of action and its role as clinical biomarkers, exosomes may become an important diagnostic and treatment method for male fertility in the future.

**TABLE 6:** Summary of MSCs and exosomes research.

Cell type	Species	Administration method	Key point	Relevant references
BM-MSCs	Mice	inject into the testes	improves SSC transplantation efficiency	Kadam et al. (2018)
BM-MSCs	Rat	inject into the testes	improve fertility of azoospermic rats	Badawy et al. (2020)
BM-MSCs	Mice	co-culture with SSCs	improved *in vitro* spermatogenesis	Önen et al. (2023)
BM-MSCs	Rat	intravenous injection	restore the male reproductive system after Dox treatment	Abdelaziz et al. (2019)
BM-MSCs	Rat	intravenous injection	reverse Cadmium induced testicular injury	Elbaghdady et al. (2018)
BM-MSCs	Rat	intravenous injection	reduce cisplatin induced gonad toxicity	Sherif et al. (2018)
ADSCs	Rat	inject into the testes	benefit to testicular torsion-induced infertility	Hsiao et al. (2015)
BM-MSCs	Rat	inject into the testes	benefit to gentamicin induced testicular dysfunctions	SM et al. (2017)
ADSCs	Human	inject into mice testes	resumption of spermatogenesis	Eliyasi et al. (2020)
AFSCs	Human	inject into rat testes		Ibrahim et al. (2021)
AMSCs	Human	inject into mice testes		Qian et al. (2020)
ADSCs	Human	inject into rat testes		Siregar et al. (2021)
ADSCs	Human	inject into rat testes	improve the damage of cisplatin to testes	Meligy et al. (2019)
ADSCs	Human	inject into mice testes	improve the spermatogenesis of azoospermia	Ganjibakhsh et al. (2022)
ADSCs	Guinea Pig	inject into the testes		Hajihoseini et al. (2018)
ADSCs	Hamsters	inject into the testes		Karimaghai et al. (2018)
UCB-SCs	Human	inject into mice testes		Abd et al. (2017)
WJ-MSCs	Human	*In vitro*	transdifferentiate WJ-MSCs into male germ cells	Dissanayake et al. (2018)
WJ-MSCs	Human	*In vitro*	differentiate into germ-like cells	Ghaem et al. (2018)
BM-MSCs	Rat	*In vitro*	differentiate into germ-like cells	Kumar et al. (2018)
ADSCs	Human	*In vitro*	differentiate into germ-like cells	Liu et al. (2018)
BM-MSCs	Mice	*In vitro*	differentiate into SSC like cells	Behzadi et al. (2019)
ADSCs	Rat	*In vitro*	induce ADSCs into generation of MGLCs	Luo et al. (2019)
HUCPVCs	Human	*In vitro*	generation of Sertoli-like and haploid spermatid-like cells from HUCPVCs	Shlush et al. (2017)
Amniotic fluid derived exosomes	Sheep	inject into rat testes	regenerated spermatogenesis and improved sperm quality in NOA rats	Mobarak et al. (2021)
MSC CM	Human	inject into rat testes	recovery of spermatogenesis in cryptorchidism	Sagaradze et al. (2019)
BM-MSCs derived exosomes	Rat	intravenous injection	protects against testicular IRI	Zhang et al. (2018a)

Abbreviation: BM-MSCs, bone marrow derived MSCs; ADSCs, adipose derived stem cells; AFSCs, amniotic fluid-derived stem cells; AMSCs, amnion mesenchymal stem cells; UCB-SCs, umbilical cord blood-derived stem cells; WJ-MSCs, Wharton’s jelly-derived mesenchymal stem cells; MGLCs, male germ-like cell; HUCPVCs, human umbilical cord-derived perivascular cells; NOA, non-obstructive azoospermia; CM: conditioned medium; IRI, ischemia-reperfusion injury.

## 5 Conclusion and discussion

The effects of drugs on male germ cells can affect testosterone production, spermatogenesis, sperm capacitation, alter genetic material of germ cells, increase oxidative stress, and regulate signaling pathways. Most drugs have negative effects on male germ cells. Although germ cells can recover after most drugs are discontinued, the negative impact of drugs on germ cells should also be taken into consideration when choosing drugs for clinical use. Drugs with obvious toxicity to germ cells should be used with caution. More research should be invested in drugs that need to be taken long-term to maintain treatment, in order to clarify their mechanisms of action and explore how to cope with the damage to germ cells caused by these drugs.

Stem cell therapy and exosomes therapy may be a new type of treatment for iatrogenic male infertility or male infertility caused by diseases in the future. The advantages of exosomes application include avoiding the degradation of active ingredients and the drawbacks of cell therapy, but their specific molecular mechanisms of action still need to be further explored. The advantage of stem cell therapy lies in self-renewal and the ability to differentiate into other special cell types. The application of different types of stem cells in the treatment of male infertility has different difficulties that need to be overcome. Therefore, while exploring its mechanism of action and applying it to regenerative medicine, we may also consider the personalized choice of its treatment scheme and the safety of its application.

## References

[B1] Abd A. S. Pasha H. F. Abdelrahman A. A. Mazen N. F. (2017). Molecular effect of human umbilical cord blood cd34-positive and cd34-negative stem cells and their conjugate in azoospermic mice. Mol. Cell. Biochem. 428 (1-2), 179–191. 10.1007/s11010-016-2928-2 28120211

[B2] Abdal D. A. Lee S. B. Kim K. Lim K. M. Jeon T. I. Seok J. (2019). Production of mesenchymal stem cells through stem cell reprogramming. Int. J. Mol. Sci. 20 (8), 1922. 10.3390/ijms20081922 31003536 PMC6514654

[B3] Abdelaal N. E. Tanga B. M. Abdelgawad M. Allam S. Fathi M. Saadeldin I. M. (2021). Cellular therapy via spermatogonial stem cells for treating impaired spermatogenesis, non-obstructive azoospermia. Cells 10 (7), 1779. 10.3390/cells10071779 34359947 PMC8304133

[B4] Abdelaziz M. H. Salah E. E. El-Dakdoky M. H. Ahmed T. A. (2019). The impact of mesenchymal stem cells on doxorubicin-induced testicular toxicity and progeny outcome of male prepubertal rats. Birth Defects Res. 111 (13), 906–919. 10.1002/bdr2.1535 31210400

[B5] Abdulwahab D. K. Ibrahim W. W. Abd E. R. Abdel-Latif H. A. Abdelkader N. F. (2021). Grape seed extract improved the fertility-enhancing effect of atorvastatin in high-fat diet-induced testicular injury in rats: involvement of antioxidant and anti-apoptotic effects. J. Pharm. Pharmacol. 73 (3), 366–376. 10.1093/jpp/rgaa002 33793875

[B6] Abu-Halima M. Ludwig N. Hart M. Leidinger P. Backes C. Keller A. (2016). Altered micro-ribonucleic acid expression profiles of extracellular microvesicles in the seminal plasma of patients with oligoasthenozoospermia. Fertil. Steril. 106 (5), 1061–1069. 10.1016/j.fertnstert.2016.06.030 27424049

[B7] Abu-Risha S. E. Mousa M. A. Elsisi A. E. (2022). Protective role of irbesartan against cyclophosphamide-induced testicular damage in rats via up-regulating ppar-γ signaling and ameliorating nf-κb/nlrp3/il-18 inflammatory axis. Life Sci. 289, 120218. 10.1016/j.lfs.2021.120218 34890588

[B8] Adaramoye O. A. Adesanoye O. A. Adewumi O. M. Akanni O. (2012). Studies on the toxicological effect of nevirapine, an antiretroviral drug, on the liver, kidney and testis of male wistar rats. Hum. Exp. Toxicol. 31 (7), 676–685. 10.1177/0960327111424304 22027508

[B9] Adaramoye O. A. Akanni O. O. Adewumi O. M. Owumi S. E. (2015). Lopinavir/ritonavir, an antiretroviral drug, lowers sperm quality and induces testicular oxidative damage in rats. Tokai J. Exp. Clin. Med. 40 (2), 51–57. 26150184

[B10] Agarwal A. Baskaran S. Parekh N. Cho C. L. Henkel R. Vij S. (2021). Male infertility. Lancet 397 (10271), 319–333. 10.1016/S0140-6736(20)32667-2 33308486

[B11] Akasheh G. Sirati L. Noshad K. A. Sepehrmanesh Z. (2014). Comparison of the effect of sertraline with behavioral therapy on semen parameters in men with primary premature ejaculation. Urology 83 (4), 800–804. 10.1016/j.urology.2013.12.004 24529582

[B12] Ali W. Bian Y. Ali H. Sun J. Zhu J. Ma Y. (2023). Cadmium-induced impairment of spermatozoa development by reducing exosomal-mvbs secretion: A novel pathway. Aging (Albany NY) 15 (10), 4096–4107. 10.18632/aging.204675 37220720 PMC10258001

[B13] Alsemeh A. E. Ahmed M. M. Fawzy A. Samy W. Tharwat M. Rezq S. (2022). Vitamin e rescues valproic acid-induced testicular injury in rats: role of autophagy. Life Sci. 296, 120434. 10.1016/j.lfs.2022.120434 35227771

[B14] Amiri N. Mohammadi P. Allahgholi A. Salek F. Amini E. (2023). The potential of sertoli cells (scs) derived exosomes and its therapeutic efficacy in male reproductive disorders. Life Sci. 312, 121251. 10.1016/j.lfs.2022.121251 36463941

[B15] Aruna N. Krishnamurthy N. B. (1986). Mutagenic evaluation of propranolol in somatic and germ cells of mice. Mutat. Res.-Fundam. Mol. Mech. Mutagen. 173 (3), 207–210. 10.1016/0165-7992(86)90037-0 3951470

[B16] Atli O. Baysal M. Aydogan-Kilic G. Kilic V. Ucarcan S. Karaduman B. (2017). Sertraline-induced reproductive toxicity in male rats: evaluation of possible underlying mechanisms. Asian J. Androl. 19 (6), 672–679. 10.4103/1008-682X.192637 27976631 PMC5676427

[B17] Attia S. M. Bakheet S. A. (2013). Citalopram at the recommended human doses after long-term treatment is genotoxic for male germ cell. Food Chem. Toxicol. 53, 281–285. 10.1016/j.fct.2012.11.051 23232079

[B18] Aydin S. Inci O. Alagöl B. (1995). The role of arginine, indomethacin and kallikrein in the treatment of oligoasthenospermia. Int. Urol. Nephrol. 27 (2), 199–202. 10.1007/BF02551320 7591579

[B19] Badawy A. A. El-Magd M. A. AlSadrah S. A. Alruwaili M. M. (2020). Altered expression of some mirnas and their target genes following mesenchymal stem cell treatment in busulfan-induced azoospermic rats. Gene 737, 144481. 10.1016/j.gene.2020.144481 32070749

[B20] Bagoji I. B. Hadimani G. A. Yendigeri S. M. Das K. K. (2017). Sub-chronic indomethacin treatment and its effect on the male reproductive system of albino rats: possible protective role of black tea extract. J. Basic Clin. Physiol. Pharmacol. 28 (3), 201–207. 10.1515/jbcpp-2016-0168 28222030

[B21] Bailey J. L. (2010). Factors regulating sperm capacitation. Syst. Biol. Reprod. Med. 56 (5), 334–348. 10.3109/19396368.2010.512377 20849222

[B22] Balistreri C. R. De Falco E. Bordin A. Maslova O. Koliada A. Vaiserman A. (2020). Stem cell therapy: old challenges and new solutions. Mol. Biol. Rep. 47 (4), 3117–3131. 10.1007/s11033-020-05353-2 32128709

[B23] Barbosa M. G. Jorge B. C. Stein J. Santos F. D. Barreto A. Reis A. (2020). Pre-pubertal exposure to ibuprofen impairs sperm parameters in male adult rats and compromises the next generation. J. Toxicol. Env. Health Part A. 83 (15-16), 559–572. 10.1080/15287394.2020.1786483 32615883

[B24] Barkay J. Harpaz-Kerpel S. Ben-Ezra S. Gordon S. Zuckerman H. (1984). The prostaglandin inhibitor effect of antiinflammatory drugs in the therapy of male infertility. Fertil. Steril. 42 (3), 406–411. 10.1016/s0015-0282(16)48081-7 6088300

[B25] Baskaran S. Panner S. M. Agarwal A. (2020). Exosomes of male reproduction. Advan. Clin. Chem. 95, 149–163. 10.1016/bs.acc.2019.08.004 32122522

[B26] Baysal M. Ilgin S. Kilic G. Kilic V. Ucarcan S. Atli O. (2017). Reproductive toxicity after levetiracetam administration in male rats: evidence for role of hormonal status and oxidative stress. PLoS One 12 (4), e0175990. 10.1371/journal.pone.0175990 28419133 PMC5395212

[B27] Behzadi F. S. Mazaheri Z. Ghorbanmehr N. Movahedin M. Behzadi F. M. Gholampour M. A. (2019). Analysis of mirna-17 and mirna-146 expression during differentiation of spermatogonial stem like cells derived from mouse bone marrow mesenchymal stem cells. Int. J. Mol. Cell. Med. 8 (1), 14–23. 10.22088/IJMCM.BUMS.8.1.14 32195202 PMC7073265

[B28] Bernini G. P. Brogi G. Argenio G. F. Moretti A. Salvetti A. (1998). Effects of long-term pravastatin treatment on spermatogenesis and on adrenal and testicular steroidogenesis in male hypercholesterolemic patients. J. Endocrinol. Investig. 21 (5), 310–317. 10.1007/BF03350334 9648053

[B29] Brinster R. L. Zimmermann J. W. (1994). Spermatogenesis following male germ-cell transplantation. Proc. Natl. Acad. Sci. U. S. A. 91 (24), 11298–11302. 10.1073/pnas.91.24.11298 7972053 PMC45218

[B30] Cai T. Mondaini N. Mazzoli S. Bartoletti R. (2008). A possible negative effect of co-administered amlodipine and atorvastatin on semen volume and spermatozoa in men. J. Pharm. Pharmacol. 60 (11), 1431–1432. 10.1211/jpp/60.11.0002 18957162

[B31] Candenas L. Chianese R. (2020). Exosome composition and seminal plasma proteome: A promising source of biomarkers of male infertility. Int. J. Mol. Sci. 21 (19), 7022. 10.3390/ijms21197022 32987677 PMC7583765

[B32] Carvalho R. K. Rocha T. L. Fernandes F. H. Gonçalves B. B. Souza M. R. Araújo A. A. (2022). Decreasing sperm quality in mice subjected to chronic cannabidiol exposure: new insights of cannabidiol-mediated male reproductive toxicity. Chem.-Biol. Interact. 351, 109743. 10.1016/j.cbi.2021.109743 34774840

[B33] Carvalho R. K. Santos M. L. Souza M. R. Rocha T. L. Guimarães F. S. Anselmo-Franci J. A. (2018). Chronic exposure to cannabidiol induces reproductive toxicity in male swiss mice. J. Appl. Toxicol. 38 (9), 1215–1223. 10.1002/jat.3631 29766538

[B34] Chen Q. Deng T. Han D. (2016). Testicular immunoregulation and spermatogenesis. Semin. Cell. Dev. Biol. 59, 157–165. 10.1016/j.semcdb.2016.01.019 26805443

[B35] Chen X. Zheng Y. Lei A. Zhang H. Niu H. Li X. (2020). Early cleavage of preimplantation embryos is regulated by trna(gln-ttg)-derived small rnas present in mature spermatozoa. J. Biol. Chem. 295 (32), 10885–10900. 10.1074/jbc.RA120.013003 32487749 PMC7415976

[B36] Chengelis C. P. Dodd D. C. Kotsonis F. N. (1986). Testicular toxicity of a novel 1,4-benzodiazepine (sc-32855) in rats and dogs. Res. Commun. Chem. Pathol. Pharmacol. 51 (1), 23–36. 3952369

[B37] Conte D. Nordio M. Romanelli F. Manganelli F. Giovenco P. Dondero F. (1985). Role of seminal prostaglandins in male fertility. Ii. Effects of prostaglandin synthesis inhibition on spermatogenesis in man. J. Endocrinol. Investig. 8 (4), 289–291. 10.1007/BF03348497 3840817

[B38] Cook P. S. Notelovitz M. Kalra P. S. Kalra S. P. (1979). Effect of diazepam on serum testosterone and the ventral prostate gland in male rats. Arch. Androl. 3 (1), 31–35. 10.3109/01485017908985045 384947

[B39] Cui X. Long C. Zhu J. Tian J. (2017). Protective effects of fluvastatin on reproductive function in obese male rats induced by high-fat diet through enhanced signaling of mtor. Cell. Physiol. Biochem. 41 (2), 598–608. 10.1159/000457881 28214901

[B40] Darwish H. A. Arab H. H. Abdelsalam R. M. (2014). Chrysin alleviates testicular dysfunction in adjuvant arthritic rats via suppression of inflammation and apoptosis: comparison with celecoxib. Toxicol. Appl. Pharmacol. 279 (2), 129–140. 10.1016/j.taap.2014.05.018 24932515

[B41] de Oliveira W. M. de Sá I. R. de Torres S. M. de Morais R. N. Andrade A. M. Maia F. C. (2013). Perinatal exposure to fluoxetine via placenta and lactation inhibits the testicular development in male rat offspring. Syst. Biol. Reprod. Med. 59 (5), 244–250. 10.3109/19396368.2013.796021 23651434

[B42] De Turner E. Aparicio N. J. Turner D. Schwarzstein L. (1978). Effect of two phosphodiesterase inhibitors, cyclic adenosine 3':5'-monophosphate, and a beta-blocking agent on human sperm motility. Fertil. Steril. 29 (3), 328–331. 10.1016/s0015-0282(16)43161-4 205445

[B43] Deng C. Xie Y. Zhang C. Ouyang B. Chen H. Lv L. (2019). Urine-derived stem cells facilitate endogenous spermatogenesis restoration of busulfan-induced nonobstructive azoospermic mice by paracrine exosomes. Stem Cells Dev. 28 (19), 1322–1333. 10.1089/scd.2019.0026 31311428

[B44] Diao L. Turek P. J. John C. M. Fang F. Reijo P. R. (2022). Roles of spermatogonial stem cells in spermatogenesis and fertility restoration. Front. Endocrinol. 13, 895528. 10.3389/fendo.2022.895528 PMC9135128 35634498

[B45] Diemer T. Desjardins C. (1999). Developmental and genetic disorders in spermatogenesis. Hum. Reprod. Update. 5 (2), 120–140. 10.1093/humupd/5.2.120 10336017

[B46] Dissanayake D. Patel H. Wijesinghe P. S. (2018). Differentiation of human male germ cells from wharton's jelly-derived mesenchymal stem cells. Clin. Exp. Reprod. Med.-CERM. 45 (2), 75–81. 10.5653/cerm.2018.45.2.75 29984207 PMC6030615

[B47] Dobs A. S. Miller S. Neri G. Weiss S. Tate A. C. Shapiro D. R. (2000). Effects of simvastatin and pravastatin on gonadal function in male hypercholesterolemic patients. Metab.-Clin. Exp. 49 (1), 115–121. 10.1016/s0026-0495(00)90938-7 10647074

[B48] Dong L. Gul M. Hildorf S. Pors S. E. Kristensen S. G. Hoffmann E. R. (2019). Xeno-free propagation of spermatogonial stem cells from infant boys. Int. J. Mol. Sci. 20 (21), 5390. 10.3390/ijms20215390 31671863 PMC6862004

[B49] Drobnis E. Z. Nangia A. K. (2017). Cardiovascular/pulmonary medications and male reproduction. Adv. Exp. Med. Biol. 1034, 103–130. 10.1007/978-3-319-69535-8_9 29256129

[B50] Duca Y. Di Cataldo A. Russo G. Cannata E. Burgio G. Compagnone M. (2019). Testicular function of childhood cancer survivors: who is worse? J. Clin. Med. 8 (12), 2204. 10.3390/jcm8122204 31847212 PMC6947348

[B51] Easley C. T. Phillips B. T. McGuire M. M. Barringer J. M. Valli H. Hermann B. P. (2012). Direct differentiation of human pluripotent stem cells into haploid spermatogenic cells. Cell. Rep. 2 (3), 440–446. 10.1016/j.celrep.2012.07.015 22921399 PMC3698576

[B52] Eguizabal C. Montserrat N. Vassena R. Barragan M. Garreta E. Garcia-Quevedo L. (2011). Complete meiosis from human induced pluripotent stem cells. Stem Cells 29 (8), 1186–1195. 10.1002/stem.672 21681858

[B53] Eid A. H. Abdelkader N. F. Abd E. O. Fawzy H. M. El-Denshary E. S. (2016). Carvedilol alleviates testicular and spermatological damage induced by cisplatin in rats via modulation of oxidative stress and inflammation. Arch. Pharm. Res. 39 (12), 1693–1702. 10.1007/s12272-016-0833-6 27620497

[B54] Eid A. H. Gad A. M. Fikry E. M. Arab H. H. (2019). Venlafaxine and carvedilol ameliorate testicular impairment and disrupted spermatogenesis in rheumatoid arthritis by targeting ampk/erk and pi3k/akt/mtor pathways. Toxicol. Appl. Pharmacol. 364, 83–96. 10.1016/j.taap.2018.12.014 30578887

[B55] Eikmans M. D H. A. J. Blijleven L. Meuleman T. van Beelen E. van der Hoorn M. P. (2020). Optimization of microrna acquirement from seminal plasma and identification of diminished seminal microrna-34b as indicator of low semen concentration. Int. J. Mol. Sci. 21 (11), 4089. 10.3390/ijms21114089 32521662 PMC7312420

[B56] El M. A. Ibrahim F. M. Mabrouk D. M. Ahmed K. A. Fawzy R. M. (2019). Effect of antiepileptic drug (topiramate) and cold pressed ginger oil on testicular genes expression, sexual hormones and histopathological alterations in mice. Biomed. Pharmacother. 110, 409–419. 10.1016/j.biopha.2018.11.146 30530043

[B57] El-Sayed M. G. El-Sayed M. T. Elazab A. E. S. Hafeiz M. H. El-Komy A. A. Hassan E. (1998). Effects of some beta-adrenergic blockers on male fertility parameters in rats. Dtsch. Tierarztl Wochenschr 105 (1), 10–12. 9499626

[B58] Elbaghdady H. Alwaili M. A. El-Demerdash R. S. (2018). Amelioration of cadmium-induced testes' damage in rats by the bone marrow mesenchymal stem cells. Ecotox. Environ. Safe. 148, 763–769. 10.1016/j.ecoenv.2017.10.016 29182986

[B59] Eliyasi D. M. Hemadi M. Saki G. Mohammadiasl J. Khodadadi A. (2020). Spermatogenesis recovery potentials after transplantation of adipose tissue-derived mesenchymal stem cells cultured with growth factors in experimental azoospermic mouse models. Cell. J. 21 (4), 401–409. 10.22074/cellj.2020.6055 31376321 PMC6722443

[B60] Elmslie K. S. (2004). Calcium channel blockers in the treatment of disease. J. Neurosci. Res. 75 (6), 733–741. 10.1002/jnr.10872 14994334

[B61] Erdemir F. Atilgan D. Firat F. Markoc F. Parlaktas B. S. Sogut E. (2014). The effect of sertraline, paroxetine, fluoxetine and escitalopram on testicular tissue and oxidative stress parameters in rats. Int. Braz J. Urol. 40 (1), 100–108. 10.1590/S1677-5538.IBJU.2014.01.15 24642156

[B62] Esmail M. Kandeil M. El-Zanaty A. M. Abdel-Gabbar M. (2020). The ameliorative effect of atorvastatin on serum testosterone and testicular oxidant/antioxidant system of hfd-fed male albino rats. F1000Res 9, 1300. 10.12688/f1000research.25926.1 33628436 PMC7876586

[B63] Farsani B. E. Karimi S. Mansouri E. (2018). Pravastatin attenuates testicular damage induced by doxorubicin - a stereological and histopatological study. J. Basic Clin. Physiol. Pharmacol. 30 (1), 103–109. 10.1515/jbcpp-2018-0073 30530881

[B64] Fayomi A. P. Peters K. Sukhwani M. Valli-Pulaski H. Shetty G. Meistrich M. L. (2019). Autologous grafting of cryopreserved prepubertal rhesus testis produces sperm and offspring. Science 363 (6433), 1314–1319. 10.1126/science.aav2914 30898927 PMC6598202

[B65] Fazeli Z. Abedindo A. Omrani M. D. Ghaderian S. M. H. (2018). Mesenchymal stem cells (mscs) therapy for recovery of fertility: A systematic review. Stem Cell. Rev. Rep. 14 (1), 1–12. 10.1007/s12015-017-9765-x 28884412

[B66] Fode M. Joensen U. N. Wiborg M. H. Fojecki G. Jørgensen N. Jensen C. (2021). Diagnosis and treatment of male infertility. Ugeskr. Laeger 183 (2), V07200565. 33491636

[B67] Fogari R. Preti P. Derosa G. Marasi G. Zoppi A. Rinaldi A. (2002). Effect of antihypertensive treatment with valsartan or atenolol on sexual activity and plasma testosterone in hypertensive men. Eur. J. Clin. Pharmacol. 58 (3), 177–180. 10.1007/s00228-002-0456-3 12107602

[B68] Foresta C. Mioni R. Rossato M. Varotto A. Zorzi M. (1991). Evidence for the involvement of sperm angiotensin converting enzyme in fertilization. Int. J. Androl. 14 (5), 333–339. 10.1111/j.1365-2605.1991.tb01101.x 1665481

[B69] Fraser L. R. (1992). Requirements for successful mammalian sperm capacitation and fertilization. Arch. Pathol. Lab. Med. 116 (4), 345–350. 1558471

[B70] Gabrielsen J. S. Tanrikut C. (2016). Chronic exposures and male fertility: the impacts of environment, diet, and drug use on spermatogenesis. Andrology 4 (4), 648–661. 10.1111/andr.12198 27230702

[B71] Ganjibakhsh M. Mehraein F. Koruji M. Bashiri Z. (2022). The therapeutic potential of adipose tissue-derived mesenchymal stromal cells in the treatment of busulfan-induced azoospermic mice. J. Assist. Reprod. Genet. 39 (1), 153–163. 10.1007/s10815-021-02309-8 34519944 PMC8866597

[B72] Gao H. Cao H. Li Z. Li L. Guo Y. Chen Y. (2023). Exosome-derived small rnas in mouse sertoli cells inhibit spermatogonial apoptosis. Theriogenology 200, 155–167. 10.1016/j.theriogenology.2023.02.011 36806925

[B73] Gao T. Zhao X. Liu C. Shao B. Zhang X. Li K. (2018). Somatic angiotensin i-converting enzyme regulates self-renewal of mouse spermatogonial stem cells through the mitogen-activated protein kinase signaling pathway. Stem Cells Dev. 27 (15), 1021–1032. 10.1089/scd.2017.0287 29792376

[B74] Ghaem M. R. Mirzapour T. Bayrami A. (2018). Differentiation of mesenchymal stem cells to germ-like cells under induction of sertoli cell-conditioned medium and retinoic acid. Andrologia 50 (3), e12887. 10.1111/and.12887 28944567

[B75] Ghasemzadeh-Hasankolaei M. Batavani R. Eslaminejad M. B. Sayahpour F. (2016). Transplantation of autologous bone marrow mesenchymal stem cells into the testes of infertile male rats and new germ cell formation. Int. J. Stem Cells. 9 (2), 250–263. 10.15283/ijsc16010 27430978 PMC5155721

[B76] Ghorbani H. Akhavanrezayat A. Jarahi L. Memar B. Amouian S. Attaranzadeh A. (2021). Effects of sertraline on spermatogenesis of male rats and its reversibility after terminating the drug. Urol. J. 18 (4), 434–438. 10.22037/uj.v18i.6458 33813731

[B77] Goossens E. Jahnukainen K. Mitchell R. T. van Pelt A. Pennings G. Rives N. (2020). Fertility preservation in boys: recent developments and new insights (†). Hum. Reprod. Open. 2020 (3), hoaa016. 10.1093/hropen/hoaa016 32529047 PMC7275639

[B78] Goossens E. Tournaye H. (2014). Male fertility preservation, where are we in 2014? Ann. Endocrinol. 75 (2), 115–117. 10.1016/j.ando.2014.03.011 24793992

[B79] Guo L. Jing J. Feng Y. M. Yao B. (2015). Tamoxifen is a potent antioxidant modulator for sperm quality in patients with idiopathic oligoasthenospermia. Int. Urol. Nephrol. 47 (9), 1463–1469. 10.1007/s11255-015-1065-2 26216675

[B80] Guo Y. Chen L. Wu D. Yu L. Sun H. Zhu Q. (2021). A comparative study of the effects of valproate and oxcarbazepine on sexual function, sperm quality, and sex hormones in males with epilepsy. Biomed. Res. Int. 2021, 6624101. 10.1155/2021/6624101 34285917 PMC8275390

[B81] Gurel C. Kuscu G. C. Buhur A. Dagdeviren M. Oltulu F. Karabay Y. N. (2019). Fluvastatin attenuates doxorubicin-induced testicular toxicity in rats by reducing oxidative stress and regulating the blood-testis barrier via mtor signaling pathway. Hum. Exp. Toxicol. 38 (12), 1329–1343. 10.1177/0960327119862006 31272229

[B82] Hajihoseini M. Mehrabani D. Vahdati A. Hosseini S. E. Tamadon A. Dianatpour M. (2018). Spermatogenesis after transplantation of adipose tissue-derived stem cells in azoospermic Guinea pigs: A histological and histomorphometric study. Galen. Med. J. 7, e1000. 10.22086/gmj.v0i0.1000 34466423 PMC8343795

[B83] Hamdi H. (2019). The preventive role of wheat germ oil against sertraline-induced testicular damage in male albino rats. Andrologia 51 (10), e13369. 10.1111/and.13369 31418462

[B84] Hong C. Y. Chiang B. N. Turner P. (1984). Calcium ion is the key regulator of human sperm function. Lancet 2 (8417-8418), 1449–1451. 10.1016/s0140-6736(84)91634-9 6151055

[B85] Horvath-Pereira B. O. Almeida G. Da S. J. L. Do N. P. Horvath P. B. Fireman J. (2023). Biomaterials for testicular bioengineering: how far have we come and where do we have to go? Front. Endocrinol. 14, 1085872. 10.3389/fendo.2023.1085872 PMC10060902 37008920

[B86] Hou M. Andersson M. Eksborg S. Söder O. Jahnukainen K. (2007). Xenotransplantation of testicular tissue into nude mice can be used for detecting leukemic cell contamination. Hum. Reprod. 22 (7), 1899–1906. 10.1093/humrep/dem085 17452397

[B87] Hsiao C. H. Ji A. T. Chang C. C. Cheng C. J. Lee L. M. Ho J. H. (2015). Local injection of mesenchymal stem cells protects testicular torsion-induced germ cell injury. Stem Cell. Res. Ther. 6 (1), 113. 10.1186/s13287-015-0079-0 26025454 PMC4449584

[B88] Iamsaard S. Sukhorum W. Arun S. Phunchago N. Uabundit N. Boonruangsri P. (2017). Valproic acid induces histologic changes and decreases androgen receptor levels of testis and epididymis in rats. Int. J. Reprod. Biomed. 15 (4), 217–224. 10.29252/ijrm.15.4.217 28835938 PMC5555039

[B89] Ibrahim H. F. Safwat S. H. Zeitoun T. M. El M. K. Medwar A. Y. (2021). The therapeutic potential of amniotic fluid-derived stem cells on busulfan-induced azoospermia in adult rats. Tissue Eng. Regen. Med. 18 (2), 279–295. 10.1007/s13770-020-00309-w 33713308 PMC8012477

[B90] Ilgin S. Kilic G. Baysal M. Kilic V. Korkut B. Ucarcan S. (2017). Citalopram induces reproductive toxicity in male rats. Birth Defects Res. 109 (7), 475–485. 10.1002/bdr2.1010 28398617

[B91] Iranloye B. O. Morakinyo A. O. Uwah J. Bello O. Daramola O. A. (2009). Effect of nifedipine on reproductive functions in male rats. Nig Q. J. Hosp. Med. 19 (3), 165–168. 10.4314/nqjhm.v19i3.54506 20836324

[B92] Ismail H. Y. Hussein S. Shaker N. A. Rizk H. Wally Y. R. (2023). Stem cell treatment trials for regeneration of testicular tissue in laboratory animals. Reprod. Sci. 30 (6), 1770–1781. 10.1007/s43032-022-01152-1 36602652 PMC10229736

[B93] Jahnukainen K. Ehmcke J. Nurmio M. Schlatt S. (2012). Autologous ectopic grafting of cryopreserved testicular tissue preserves the fertility of prepubescent monkeys that receive sterilizing cytotoxic therapy. Cancer Res. 72 (20), 5174–5178. 10.1158/0008-5472.CAN-12-1317 22902414 PMC3971428

[B94] Jahnukainen K. Hou M. Petersen C. Setchell B. Söder O. (2001). Intratesticular transplantation of testicular cells from leukemic rats causes transmission of leukemia. Cancer Res. 61 (2), 706–710. 11212272

[B95] Jiang B. Yan L. Wang X. Li E. Murphy K. Vaccaro K. (2019). Concise review: mesenchymal stem cells derived from human pluripotent cells, an unlimited and quality-controllable source for therapeutic applications. Stem Cells 37 (5), 572–581. 10.1002/stem.2964 30561809

[B96] Juneja R. Gupta I. Wali A. Sanyal S. N. Chakravarti R. N. Majumdar S. (1990). Effect of verapamil on different spermatozoal functions in Guinea pigs-a preliminary study. Contraception 41 (2), 179–187. 10.1016/0010-7824(90)90146-m 2311403

[B97] Kabel A. M. Salama S. A. Alghorabi A. A. Estfanous R. S. (2020). Amelioration of cyclosporine-induced testicular toxicity by carvedilol and/or alpha-lipoic acid: role of tgf-β1, the proinflammatory cytokines, nrf2/ho-1 pathway and apoptosis. Clin. Exp. Pharmacol. Physiol. 47 (7), 1169–1181. 10.1111/1440-1681.13281 32052493

[B98] Kadam P. Ntemou E. Baert Y. Van Laere S. Van Saen D. Goossens E. (2018). Co-transplantation of mesenchymal stem cells improves spermatogonial stem cell transplantation efficiency in mice. Stem Cell. Res. Ther. 9 (1), 317. 10.1186/s13287-018-1065-0 30463610 PMC6249754

[B99] Kar R. N. Das R. K. (1983). Induction of sperm head abnormalities in mice by three tranquilizers. Cytobios 36 (141), 45–51. 6132780

[B100] Karimaghai N. Tamadon A. Rahmanifar F. Mehrabani D. Raayat J. A. Zare S. (2018). Spermatogenesis after transplantation of adipose tissue-derived mesenchymal stem cells in busulfan-induced azoospermic hamster. Iran. J. Basic Med. Sci. 21 (7), 660–667. 10.22038/IJBMS.2018.29040.7010 30140403 PMC6098960

[B101] Khaled E. Abo E. F. El-Nesr K. A. Zanaty M. I. El-Banna H. A. El-Shahawy A. (2020). An herbal nanohybrid formula of epigallocatechin gallate-chitosan-alginate efficiently restrict the sexual dysfunction adverse effect of β-adrenergic antagonist drug: propranolol. J. Biomed. Nanotechnol. 16 (4), 505–519. 10.1166/jbn.2020.2915 32970982

[B102] Khan U. A. Aslam M. Saeed S. A. (2004). Effect of beta adrenergic antagonist on the production of testosterone by rat’s leydig cells. J. Ayub Med. Coll. Abbottabad 16 (4), 26–28. 15762058

[B103] Köhler-Samouilidis G. Schmidt-Adamopoulou B. Samouilidis S. Papaioannou N. Kotsaki-Kovatsi V. P. (1997). Effects of captopril on the male reproductive organs and various semen parameters of rabbits. Berl. Munch Tierarztl Wochenschr 110 (6), 201–205. 9290042

[B104] Kumar K. Das K. Ap M. Kumar A. Singh P. Mondal T. (2018). Rat bone marrow derived mesenchymal stem cells differentiate to germ cell like cells. Cold Spring Harbor. Germany: Cold Spring Harbor Laboratory Press. (Reprinted.

[B105] Kushwaha S. Jena G. B. (2012). Enalapril reduces germ cell toxicity in streptozotocin-induced diabetic rat: investigation on possible mechanisms. Schmiedeb. Arch. Pharmacol. 385 (2), 111–124. 10.1007/s00210-011-0707-x 22071577

[B106] Lal A. Pike J. Polley E. L. Huang S. Sanni M. Hailat T. (2022). Comparison of rna content from hydrophobic interaction chromatography-isolated seminal plasma exosomes from intrauterine insemination (iui) pregnancies. Andrologia 54 (2), e14325. 10.1111/and.14325 34837240

[B107] Latif R. Lodhi G. M. Aslam M. (2008). Effects of amlodipine on serum testosterone, testicular weight and gonado-somatic index in adult rats. J. Ayub Med. Coll. Abbottabad 20 (4), 8–10. 19999192

[B108] Lee J. H. Kim H. Kim D. H. Gye M. C. (2006). Effects of calcium channel blockers on the spermatogenesis and gene expression in peripubertal mouse testis. Arch. Androl. 52 (4), 311–318. 10.1080/01485010600664024 16728347

[B109] Leite G. de Barros J. Martins A. J. Anselmo-Franci J. A. Barbosa F. J. Kempinas W. G. (2019). Ascorbic acid supplementation ameliorates testicular hormonal signaling, sperm production and oxidative stress in male rats exposed to rosuvastatin during pre-puberty. J. Appl. Toxicol. 39 (2), 305–321. 10.1002/jat.3720 30240002

[B110] Leite G. Figueiredo T. M. Pacheco T. L. Sanabria M. Silva P. Fernandes F. H. (2017). Vitamin c partially prevents reproductive damage in adult male rats exposed to rosuvastatin during prepuberty. Food Chem. Toxicol. 109 (1), 272–283. 10.1016/j.fct.2017.09.003 28887090

[B111] Leite G. Sanabria M. Cavariani M. M. Anselmo-Franci J. A. Pinheiro P. Domeniconi R. F. (2018). Lower sperm quality and testicular and epididymal structural impairment in adult rats exposed to rosuvastatin during prepuberty. J. Appl. Toxicol. 38 (6), 914–929. 10.1002/jat.3599 29460396

[B112] Li C. Y. Jiang L. Y. Chen W. Y. Li K. Sheng H. Q. Ni Y. (2010). Cftr is essential for sperm fertilizing capacity and is correlated with sperm quality in humans. Hum. Reprod. 25 (2), 317–327. 10.1093/humrep/dep406 19923167

[B113] Li Y. Wu Q. Li X. Von Tungeln L. S. Beland F. A. Petibone D. (2022). *In vitro* effects of cannabidiol and its main metabolites in mouse and human sertoli cells. Food Chem. Toxicol. 159, 112722. 10.1016/j.fct.2021.112722 34871667 PMC10123765

[B114] Liu H. Chen M. Liu L. Ren S. Cheng P. Zhang H. (2018). Induction of human adipose-derived mesenchymal stem cells into germ lineage using retinoic acid. Cell. Reprogr. 20 (2), 127–134. 10.1089/cell.2017.0063 29620445

[B115] Liu Z. Cao K. Liao Z. Chen Y. Lei X. Wei Q. (2020). Monophosphoryl lipid a alleviated radiation-induced testicular injury through tlr4-dependent exosomes. J. Cell. Mol. Med. 24 (7), 3917–3930. 10.1111/jcmm.14978 32135028 PMC7171420

[B116] Luo Y. Xie L. Mohsin A. Ahmed W. Xu C. Peng Y. (2019). Efficient generation of male germ-like cells derived during co-culturing of adipose-derived mesenchymal stem cells with sertoli cells under retinoic acid and testosterone induction. Stem Cell. Res. Ther. 10 (1), 91. 10.1186/s13287-019-1181-5 30867048 PMC6415496

[B117] Ma Y. Ma Q. Sun Y. Chen X. (2023). The emerging role of extracellular vesicles in the testis. Hum. Reprod. 38 (3), 334–351. 10.1093/humrep/dead015 36728671

[B118] Martin-Inaraja M. Ferreira M. Taelman J. Eguizabal C. Chuva D. S. L. S. (2021). Improving *in vitro* culture of human male fetal germ cells. Cells 10 (8), 2033. 10.3390/cells10082033 34440801 PMC8393746

[B119] Martini A. C. Vincenti L. M. Santillán M. E. Stutz G. Kaplan R. Ruiz R. D. (2008). Chronic administration of nonsteroidal-antiinflammatory drugs (nsaids): effects upon mouse reproductive functions. Rev. Fac. Cien Med. Univ. Nac. Cordoba 65 (2), 41–51. 20803938

[B120] Matuszewska A. Nowak B. Niżański W. Eberhardt M. Domrazek K. Nikodem A. (2021). Long-term administration of abacavir and etravirine impairs semen quality and alters redox system and bone metabolism in growing male wistar rats. Oxidative Med. Cell. Longev. 2021, 5596090. 10.1155/2021/5596090 PMC8349296 34373766

[B121] Mazhari S. Razi M. Sadrkhanlou R. (2018). Silymarin and celecoxib ameliorate experimental varicocele-induced pathogenesis: evidences for oxidative stress and inflammation inhibition. Int. Urol. Nephrol. 50 (6), 1039–1052. 10.1007/s11255-018-1862-5 29623501

[B122] Mbah A. U. Ndukwu G. O. Ghasi S. I. Shu E. N. Ozoemena F. N. Mbah J. O. (2012). Low-dose lisinopril in normotensive men with idiopathic oligospermia and infertility: A 5-year randomized, controlled, crossover pilot study. Clin. Pharmacol. Ther. 91 (4), 582–589. 10.1038/clpt.2011.265 22378155

[B123] Means J. R. Chengelis C. P. Jasty V. (1982). Testicular toxicity induced by oral administration of sc-32855, a 1,4-benzodiazepine, in the dog. Res. Commun. Chem. Pathol. Pharmacol. 37 (2), 317–320. 6127762

[B124] Meligy F. Y. Abo E. A. Alghareeb S. M. (2019). Therapeutic effect of adipose-derived mesenchymal stem cells on cisplatin induced testicular damage in adult male albino rat. Ultrastruct. Pathol. 43 (1), 28–55. 10.1080/01913123.2019.1572256 30741078

[B125] Miyaso H. Ogawa Y. Itoh M. (2022). Microenvironment for spermatogenesis and sperm maturation. Histochem. Cell. Biol. 157 (3), 273–285. 10.1007/s00418-021-02071-z 35247091

[B126] Mobarak H. Heidarpour M. Rahbarghazi R. Nouri M. Mahdipour M. (2021). Amniotic fluid-derived exosomes improved spermatogenesis in a rat model of azoospermia. Life Sci. 274, 119336. 10.1016/j.lfs.2021.119336 33716061

[B127] Monsefi M. Fereydouni B. Rohani L. Talaei T. (2013). Mesenchymal stem cells repair germinal cells of seminiferous tubules of sterile rats. Iran. J. Reprod. Med. 11 (7), 537–544. 24639788 PMC3941348

[B128] Moraveji S. F. Esfandiari F. Sharbatoghli M. Taleahmad S. Nikeghbalian S. Shahverdi A. (2019). Optimizing methods for human testicular tissue cryopreservation and spermatogonial stem cell isolation. J. Cell. Biochem. 120 (1), 613–621. 10.1002/jcb.27419 30242874

[B129] Murphy M. B. Moncivais K. Caplan A. I. (2013). Mesenchymal stem cells: environmentally responsive therapeutics for regenerative medicine. Exp. Mol. Med. 45 (11), e54. 10.1038/emm.2013.94 24232253 PMC3849579

[B130] Naderi M. Ahangar N. Badakhshan F. Ghasemi M. Shaki F. (2021). Zinc and selenium supplement mitigated valproic acid-induced testis toxicity by modulating the oxidative redox balance in male rats. Anat. Cell. Biol. 54 (3), 387–394. 10.5115/acb.20.280 34588319 PMC8493015

[B131] Nayernia K. Nolte J. Michelmann H. W. Lee J. H. Rathsack K. Drusenheimer N. (2006). *In vitro*-differentiated embryonic stem cells give rise to male gametes that can generate offspring mice. Dev. Cell. 11 (1), 125–132. 10.1016/j.devcel.2006.05.010 16824959

[B132] Nelson L. Cariello L. (1989). Adrenergic stimulation of sea urchin sperm cells. Gamete Res. 24 (3), 291–302. 10.1002/mrd.1120240306 2574704

[B133] Neto F. T. Bach P. V. Najari B. B. Li P. S. Goldstein M. (2016). Spermatogenesis in humans and its affecting factors. Semin. Cell. Dev. Biol. 59, 10–26. 10.1016/j.semcdb.2016.04.009 27143445

[B134] Nickkholgh B. Mizrak S. C. van Daalen S. K. Korver C. M. Sadri-Ardekani H. Repping S. (2014). Genetic and epigenetic stability of human spermatogonial stem cells during long-term culture. Fertil. Steril. 102 (6), 1700–1707. 10.1016/j.fertnstert.2014.08.022 25256932

[B135] Nishimura T. Sakai M. Yonezawa H. (2000). Effects of valproic acid on fertility and reproductive organs in male rats. J. Toxicol. Sci. 25 (2), 85–93. 10.2131/jts.25.85 10845186

[B136] Nusier M. K. Bataineh H. N. Daradka H. M. (2007). Adverse effects of propranolol on reproductive function in adult male mice. Pak J. Biol. Sci. 10 (16), 2728–2731. 10.3923/pjbs.2007.2728.2731 19070091

[B137] Okeahialam B. N. Amadi K. Ameh A. S. (2006). Effect of lisnopril, an angiotensin converting enzyme (ace) inhibitor on spermatogenesis in rats. Arch. Androl. 52 (3), 209–213. 10.1080/01485010500398012 16574603

[B138] Önen S. Atik A. C. Gizer M. Köse S. Yaman Ö. Külah H. (2023). A pumpless monolayer microfluidic device based on mesenchymal stem cell-conditioned medium promotes neonatal mouse *in vitro* spermatogenesis. Stem Cell. Res. Ther. 14 (1), 127. 10.1186/s13287-023-03356-x 37170113 PMC10173473

[B139] Otoom S. Batieneh H. Hassan Z. Daoud A. (2004). Effects of long-term use topiramate on fertility and growth parameter in adult male rats. Neuroendocrinol. Lett. 25 (5), 351–355. 15580169

[B140] Ourique G. M. Pês T. S. Saccol E. M. Finamor I. A. Glanzner W. G. Baldisserotto B. (2016a). Resveratrol prevents oxidative damage and loss of sperm motility induced by long-term treatment with valproic acid in wistar rats. Exp. Toxicol. Pathol. 68 (8), 435–443. 10.1016/j.etp.2016.07.001 27432062

[B141] Ourique G. M. Saccol E. M. Pês T. S. Glanzner W. G. Schiefelbein S. H. Woehl V. M. (2016b). Protective effect of vitamin e on sperm motility and oxidative stress in valproic acid treated rats. Food Chem. Toxicol. 95, 159–167. 10.1016/j.fct.2016.07.011 27424124

[B142] Patra P. B. Wadsworth R. M. (1991). Quantitative evaluation of spermatogenesis in mice following chronic exposure to cannabinoids. Andrologia 23 (2), 151–156. 10.1111/j.1439-0272.1991.tb02520.x 1659250

[B143] Pendleton C. Li Q. Chesler D. A. Yuan K. Guerrero-Cazares H. Quinones-Hinojosa A. (2013). Mesenchymal stem cells derived from adipose tissue vs bone marrow: *in vitro* comparison of their tropism towards gliomas. PLoS One 8 (3), e58198. 10.1371/journal.pone.0058198 23554877 PMC3595264

[B144] Peris S. Solanes D. Peña A. Enric-Rodríguez-Gil J. Riga T. (2000). Ion-mediated resistance to osmotic changes of ram spermatozoa: the role of amiloride and ouabain. Theriogenology 54 (9), 1453–1467. 10.1016/s0093-691x(00)00467-2 11191869

[B145] Pietzak E. R. Tasian G. E. Tasian S. K. Brinster R. L. Carlson C. Ginsberg J. P. (2015). Histology of testicular biopsies obtained for experimental fertility preservation protocol in boys with cancer. J. Urol. 194 (5), 1420–1424. 10.1016/j.juro.2015.04.117 26032139 PMC4615387

[B146] Portela J. de Winter-Korver C. M. van Daalen S. Meißner A. de Melker A. A. Repping S. (2019). Assessment of fresh and cryopreserved testicular tissues from (pre)pubertal boys during organ culture as a strategy for *in vitro* spermatogenesis. Hum. Reprod. 34 (12), 2443–2455. 10.1093/humrep/dez180 31858131 PMC6936721

[B147] Prasad P. Ogawa S. Parhar I. S. (2015). Serotonin reuptake inhibitor citalopram inhibits gnrh synthesis and spermatogenesis in the male zebrafish. Biol. Reprod. 93 (4), 102. 10.1095/biolreprod.115.129965 26157069

[B148] Purvis K. Tollefsrud A. Rui H. Haug E. Norseth J. Viksmoen L. (1992). Short-term effects of treatment with simvastatin on testicular function in patients with heterozygous familial hypercholesterolaemia. Eur. J. Clin. Pharmacol. 42 (1), 61–64. 10.1007/BF00314921 1541317

[B149] Qian C. Meng Q. Lu J. Zhang L. Li H. Huang B. (2020). Human amnion mesenchymal stem cells restore spermatogenesis in mice with busulfan-induced testis toxicity by inhibiting apoptosis and oxidative stress. Stem Cell. Res. Ther. 11 (1), 290. 10.1186/s13287-020-01803-7 32678012 PMC7367397

[B150] Qiu J. Ma H. Pei Y. (2023). Efficacy of cellular therapy for azoospermia in animal models: A systematic review and meta-analysis. Exp. Clin. Transpl. 21 (3), 197–210. 10.6002/ect.2022.0327 36987796

[B151] Qu Y. He Y. Meng B. Zhang X. Ding J. Kou X. (2022). Apoptotic vesicles inherit sox2 from pluripotent stem cells to accelerate wound healing by energizing mesenchymal stem cells. Acta Biomater. 149, 258–272. 10.1016/j.actbio.2022.07.009 35830925

[B152] Rahban R. Rehfeld A. Schiffer C. Brenker C. Egeberg P. D. Wang T. (2021). The antidepressant sertraline inhibits catsper ca2+ channels in human sperm. Hum. Reprod. 36 (10), 2638–2648. 10.1093/humrep/deab190 34486673 PMC8450872

[B153] Rai U. Nirmal B. K. (2003). Significance of regional difference in ion concentrations in lizard, hemidactylus flaviviridis (rüppell): assessment of ionic influence on sperm motility *in vitro* . Indian J. Exp. Biol. 41 (12), 1431–1435. 15320497

[B154] Ramos A. C. H D. S. A. Silveira K. M. Kiss A. C. Mesquita S. F. Gerardin D. C. (2015). Maternal treatment with fluoxetine promotes testicular alteration in male rat pups. Reprod. Fertil. Dev. 10.1071/RD14199 25582582

[B155] Ramzy M. M. El-Sheikh A. A. Kamel M. Y. Abdelwahab S. A. Morsy M. A. (2014). Mechanism of testicular protection of carvedilol in streptozotocin-induced diabetic rats. Indian J. Pharmacol. 46 (2), 161–165. 10.4103/0253-7613.129307 24741186 PMC3987183

[B156] Rosen R. C. Kostis J. B. Jekelis A. W. (1988). Beta-blocker effects on sexual function in normal males. Arch. Sex. Behav. 17 (3), 241–255. 10.1007/BF01541742 2900627

[B157] Røste L. S. TaubØll E. Berner A. Berg K. A. Aleksandersen M. Gjerstad L. (2001). Morphological changes in the testis after long-term valproate treatment in male wistar rats. Seizure 10 (8), 559–565. 10.1053/seiz.2001.0545 11792156

[B158] Sabapathy V. Kumar S. (2016). Hipsc-derived imscs: nextgen mscs as an advanced therapeutically active cell resource for regenerative medicine. J. Cell. Mol. Med. 20 (8), 1571–1588. 10.1111/jcmm.12839 27097531 PMC4956943

[B159] Sagaradze G. D. Basalova N. A. Kirpatovsky V. I. Ohobotov D. A. Grigorieva O. A. Balabanyan V. Y. (2019). Application of rat cryptorchidism model for the evaluation of mesenchymal stromal cell secretome regenerative potential. Biomed. Pharmacother. 109, 1428–1436. 10.1016/j.biopha.2018.10.174 30551394

[B160] Saha L. Bhargava V. K. Garg S. K. Majumdar S. (2000a). Effect of nimodipine on male reproductive functions in rats. Indian J. Physiol. Pharmacol. 44 (4), 449–455. 11214500

[B161] Saha L. Garg S. K. Bhargava V. K. Mazumdar S. (2000b). Role of angiotensin-converting enzyme inhibitor, lisinopril, on spermatozoal functions in rats. Methods Find. Exp. Clin. Pharmacol. 22 (3), 159–162. 10.1358/mf.2000.22.3.796102 10893698

[B162] Sakr H. F. Abbas A. M. Elsamanoudy A. Z. Ghoneim F. M. (2015). Effect of fluoxetine and resveratrol on testicular functions and oxidative stress in a rat model of chronic mild stress-induced depression. J. Physiol. Pharmacol. 66 (4), 515–527. 26348076

[B163] Saksena S. K. Lau I. F. Bartke A. Chang M. C. (1975). Effect of indomethacin on blood plasma levels of lh and testosterone in male rats. J. Reprod. Fertil. 42 (2), 311–317. 10.1530/jrf.0.0420311 1117445

[B164] Salek F. Baharara J. Shahrokhabadi K. N. Amini E. (2021). The guardians of germ cells; Sertoli-derived exosomes against electromagnetic field-induced oxidative stress in mouse spermatogonial stem cells. Theriogenology 173, 112–122. 10.1016/j.theriogenology.2021.08.001 34371438

[B165] Salem H. M. Mohamed T. K. Badary D. M. Ahmed M. W. Mohamed M. E. (2020). Pregabalin administration and withdrawal affect testicular structure and functions in rats. Andrologia 52 (11), e13808. 10.1111/and.13808 32882064

[B166] Sanbuissho A. Terada S. Suzuki K. Masuda N. Teranishi M. Masuda H. (1995). Male reproductive toxicity study of nitrazepam in rats. J. Toxicol. Sci. 20 (3), 319–328. 10.2131/jts.20.319 8667456

[B167] Sanou I. van Maaren J. Eliveld J. Lei Q. Meißner A. de Melker A. A. (2022). Spermatogonial stem cell-based therapies: taking preclinical research to the next level. Front. Endocrinol. 13, 850219. 10.3389/fendo.2022.850219 PMC9013905 35444616

[B168] Serrano J. B. Tabeling N. C. de Winter-Korver C. M. van Daalen S. van Pelt A. Mulder C. L. (2023). Sperm dna methylation is predominantly stable in mice offspring born after transplantation of long-term cultured spermatogonial stem cells. Clin. Epigenetics. 15 (1), 58. 10.1186/s13148-023-01469-x 37029425 PMC10080964

[B169] Shalaby M. A. El-Zorba H. Y. Kamel G. M. (2004). Effect of alpha-tocopherol and simvastatin on male fertility in hypercholesterolemic rats. Pharmacol. Res. 50 (2), 137–142. 10.1016/j.phrs.2003.10.013 15177301

[B170] Sharma P. Kaushal N. Saleth L. R. Ghavami S. Dhingra S. Kaur P. (2023). Oxidative stress-induced apoptosis and autophagy: balancing the contrary forces in spermatogenesis. Biochim. Biophys. Acta-Mol. Basis Dis. 1869 (6), 166742. 10.1016/j.bbadis.2023.166742 37146914

[B171] Sharma S. Wistuba J. Pock T. Schlatt S. Neuhaus N. (2019). Spermatogonial stem cells: updates from specification to clinical relevance. Hum. Reprod. Update. 25 (3), 275–297. 10.1093/humupd/dmz006 30810745

[B172] Sherif I. O. Sabry D. Abdel-Aziz A. Sarhan O. M. (2018). The role of mesenchymal stem cells in chemotherapy-induced gonadotoxicity. Stem Cell. Res. Ther. 9 (1), 196. 10.1186/s13287-018-0946-6 30021657 PMC6052634

[B173] Shlush E. Maghen L. Swanson S. Kenigsberg S. Moskovtsev S. Barretto T. (2017). *In vitro* generation of sertoli-like and haploid spermatid-like cells from human umbilical cord perivascular cells. Stem Cell. Res. Ther. 8 (1), 37. 10.1186/s13287-017-0491-8 28202061 PMC5312448

[B174] Shokry D. A. El N. N. Yassa H. D. Gaber S. S. Batiha G. E. Welson N. N. (2020). Pregabalin induced reproductive toxicity and body weight changes by affecting caspase3 and leptin expression: protective role of wheat germ oil. Life Sci. 260, 118344. 10.1016/j.lfs.2020.118344 32853651

[B175] Simon C. Greening D. W. Bolumar D. Balaguer N. Salamonsen L. A. Vilella F. (2018). Extracellular vesicles in human reproduction in health and disease. Endocr. Rev. 39 (3), 292–332. 10.1210/er.2017-00229 29390102

[B176] Siregar S. Noegroho B. S. Adriansjah R. Mustafa A. Bonar A. (2021). The effect of intratesticular injection of human adipose-derived mesenchymal cell on testicular oxidative stress and spermatogenesis process in the varicocele rat model. Res. Rep. Urol. 13, 759–765. 10.2147/RRU.S330634 34676179 PMC8519792

[B177] Sm M. Nm E. Sf M. Ni H. Sy S. Mr B. (2017). Effect of stem cell therapy on gentamicin induced testicular dysfunction in rats. J. Health & Med. Inf. 08 (03). 10.4172/2157-7420.1000263

[B178] Smarr M. M. Kannan K. Chen Z. Kim S. Buck L. G. (2017). Male urinary paracetamol and semen quality. Andrology 5 (6), 1082–1088. 10.1111/andr.12413 28853221 PMC10506067

[B179] Sojoudi K. Azizi H. Skutella T. (2023). A review of the potential of induced pluripotent stem cell-derived exosome as a novel treatment for male infertility. Biotechnol. Genet. Eng. Rev., 1–26. 10.1080/02648725.2023.2193772 36951621

[B180] Sołek P. Mytych J. Tabęcka-Łonczyńska A. Koziorowski M. (2021). Molecular consequences of depression treatment: A potential *in vitro* mechanism for antidepressants-induced reprotoxic side effects. Int. J. Mol. Sci. 22 (21), 11855. 10.3390/ijms222111855 34769286 PMC8584852

[B181] Solek P. Mytych J. Tabecka-Lonczynska A. Sowa-Kucma M. Koziorowski M. (2021). Toxic effect of antidepressants on male reproductive system cells: evaluation of possible fertility reduction mechanism. J. Physiol. Pharmacol. 72 (3). 10.26402/jpp.2021.3.06 34810289

[B182] Sukhorum W. Iamsaard S. (2017). Changes in testicular function proteins and sperm acrosome status in rats treated with valproic acid. Reproduction, Fertil. Dev. 29 (8), 1585–1592. 10.1071/RD16205 27511211

[B183] Suzuki H. Tominaga T. Kumagai H. Saruta T. (1988). Effects of first-line antihypertensive agents on sexual function and sex hormones. J. Hypertens. Suppl. 6 (4), S649–S651. 10.1097/00004872-198812040-00204 3149291

[B184] Taheri B. Soleimani M. Fekri A. S. Esmaeili E. Bazi Z. Zarghami N. (2019). Induced pluripotent stem cell-derived extracellular vesicles: A novel approach for cell-free regenerative medicine. J. Cell. Physiol. 234 (6), 8455–8464. 10.1002/jcp.27775 30478831

[B185] Taha S. Zaghloul H. S. Ali A. Rashed L. A. Sabry R. M. Gaballah I. F. (2020). Molecular and hormonal changes caused by long-term use of high dose pregabalin on testicular tissue: the role of p38 mapk, oxidative stress and apoptosis. Mol. Biol. Rep. 47 (11), 8523–8533. 10.1007/s11033-020-05894-6 33051752

[B186] Taher M. A. Zainab N. H. Anber H. (2019). Effect of diazepam on the reproductive system in male rats.

[B187] Takahashi K. Yamanaka S. (2006). Induction of pluripotent stem cells from mouse embryonic and adult fibroblast cultures by defined factors. Cell. 126 (4), 663–676. 10.1016/j.cell.2006.07.024 16904174

[B188] Tanrikut C. Feldman A. S. Altemus M. Paduch D. A. Schlegel P. N. (2010). Adverse effect of paroxetine on sperm. Fertil. Steril. 94 (3), 1021–1026. 10.1016/j.fertnstert.2009.04.039 19515367

[B189] Tektemur A. Etem Ö. E. Kaya T. N. Dayan C. S. Kılınçlı Ç. A. Tekedereli 0. (2021). Carbamazepine-induced sperm disorders can be associated with the altered expressions of testicular kcnj11/mir-let-7a and spermatozoal cftr/mir-27a. Andrologia 53 (2), e13954. 10.1111/and.13954 33372325

[B190] Thiageswaran S. Steele H. Voigt A. L. Dobrinski I. (2022). A role for exchange of extracellular vesicles in porcine spermatogonial co-culture. Int. J. Mol. Sci. 23 (9), 4535. 10.3390/ijms23094535 35562927 PMC9103065

[B191] Valli-Pulaski H. Peters K. A. Gassei K. Steimer S. R. Sukhwani M. Hermann B. P. (2019). Testicular tissue cryopreservation: 8 years of experience from a coordinated network of academic centers. Hum. Reprod. 34 (6), 966–977. 10.1093/humrep/dez043 31111889 PMC6554046

[B192] Vander B. M. Wyns C. (2018). Fertility and infertility: definition and epidemiology. Clin. Biochem. 62, 2–10. 10.1016/j.clinbiochem.2018.03.012 29555319

[B193] Vieira M. L. Hamada R. Y. Gonzaga N. I. Bacchi A. D. Barbieri M. Moreira E. G. (2013). Could maternal exposure to the antidepressants fluoxetine and st. John's wort induce long-term reproductive effects on male rats? Reprod. Toxicol. 35, 102–107. 10.1016/j.reprotox.2012.07.006 22824787

[B194] Wang A. (2021). Human induced pluripotent stem cell-derived exosomes as a new therapeutic strategy for various diseases. Int. J. Mol. Sci. 22 (4), 1769. 10.3390/ijms22041769 33578948 PMC7916646

[B195] Wang M. Liu X. Chang G. Chen Y. An G. Yan L. (2018). Single-cell rna sequencing analysis reveals sequential cell fate transition during human spermatogenesis. Cell. Stem Cell. 23 (4), 599–614. 10.1016/j.stem.2018.08.007 30174296

[B196] Whelan E. C. Yang F. Avarbock M. R. Sullivan M. C. Beiting D. P. Brinster R. L. (2022). Reestablishment of spermatogenesis after more than 20 years of cryopreservation of rat spermatogonial stem cells reveals an important impact in differentiation capacity. PLoS. Biol. 20 (5), e3001618. 10.1371/journal.pbio.3001618 35536782 PMC9089916

[B197] White D. R. Clarkson J. S. Ratnasooriya W. D. Aitken R. J. (1995). Complementary effects of propranolol and nonoxynol-9 upon human sperm motility. Contraception 52 (4), 241–247. 10.1016/0010-7824(95)00190-l 8605783

[B198] Wiger R. Hongslo J. K. Evenson D. P. De Angelis P. Schwarze P. E. Holme J. A. (1995). Effects of acetaminophen and hydroxyurea on spermatogenesis and sperm chromatin structure in laboratory mice. Reprod. Toxicol. 9 (1), 21–33. 10.1016/0890-6238(94)00052-x 8520128

[B199] Wong P. Y. Lee W. M. Tsang A. Y. (1981). The effects of sodium and amiloride on the motility of the caudal epididymal spermatozoa of the rat. Experientia 37 (1), 69–71. 10.1007/BF01965575 7202674

[B200] Wu D. Chen L. Ji F. Si Y. Sun H. (2018). The effects of oxcarbazepine, levetiracetam, and lamotrigine on semen quality, sexual function, and sex hormones in male adults with epilepsy. Epilepsia 59 (7), 1344–1350. 10.1111/epi.14450 29889310

[B201] Yakubu M. T. Atoyebi A. R. (2018). Brysocarpus coccineus (schum & thonn) root reinstates sexual competence and testicular function in paroxetine-induced sexual dysfunction in male wistar rats. Andrologia 50, e12980. 10.1111/and.12980 29468717

[B202] Yardimci A. Ulker N. Bulmus O. Kaya N. Colakoglu N. Ozcan M. (2019). Effects of long-term paroxetine or bupropion treatment on puberty onset, reproductive and feeding parameters in adolescent male rats. Andrologia 51 (6), e13268. 10.1111/and.13268 30873645

[B203] Zarandi N. P. Galdon G. Kogan S. Atala A. Sadri-Ardekani H. (2018). Cryostorage of immature and mature human testis tissue to preserve spermatogonial stem cells (sscs): A systematic review of current experiences toward clinical applications. Stem Cells Cloning Adv. Appl. 11, 23–38. 10.2147/SCCAA.S137873 PMC6039063 30013372

[B204] Zegers-Hochschild F. Adamson G. D. Dyer S. Racowsky C. de Mouzon J. Sokol R. (2017). The international glossary on infertility and fertility care, 2017. Fertil. Steril. 108 (3), 393–406. 10.1016/j.fertnstert.2017.06.005 28760517

[B205] Zhang D. Liu X. Peng J. He D. Lin T. Zhu J. (2014). Potential spermatogenesis recovery with bone marrow mesenchymal stem cells in an azoospermic rat model. Int. J. Mol. Sci. 15 (8), 13151–13165. 10.3390/ijms150813151 25062349 PMC4159785

[B206] Zhang W. Cheng Y. Guo W. Guo X. Bian J. Zhou Q. (2018b). rotective effect of bone marrow mesenchymal stem cells-derived exosomes against testicular ischemia-reperfusion injury in rats. J. South Med. Univ. 38 (8), 910–916. 10.3969/j.issn.1673-4254.2018.08.02 PMC6744034 30187884

[B207] Zhang W. Yang C. Guo W. Guo X. Bian J. Zhou Q. (2018a). Rotective effect of bone marrow mesenchymal stem cells-derived exosomes against testicular ischemia-reperfusion injury in rats. Nan Fang. Yi Ke Da Xue Xue Bao 38 (8), 910–916. 10.3969/j.issn.1673-4254.2018.08.02 30187884 PMC6744034

[B208] Zhankina R. Baghban N. Askarov M. Saipiyeva D. Ibragimov A. Kadirova B. (2021). Mesenchymal stromal/stem cells and their exosomes for restoration of spermatogenesis in non-obstructive azoospermia: A systemic review. Stem Cell. Res. Ther. 12 (1), 229. 10.1186/s13287-021-02295-9 33823925 PMC8025392

[B209] Zhao L. Yao C. Xing X. Jing T. Li P. Zhu Z. (2020). Single-cell analysis of developing and azoospermia human testicles reveals central role of sertoli cells. Nat. Commun. 11 (1), 5683. 10.1038/s41467-020-19414-4 33173058 PMC7655944

[B210] Zhao N. Sheng M. Wang X. Li Y. Farzaneh M. (2021). Differentiation of human induced pluripotent stem cells into male germ cells. Curr. Stem Cell. Res. Ther. 16 (5), 622–629. 10.2174/1574888X15666200705214223 32628592

[B211] Zhou Q. Wang M. Yuan Y. Wang X. Fu R. Wan H. (2016). Complete meiosis from embryonic stem cell-derived germ cells *in vitro* . Cell. Stem Cell. 18 (3), 330–340. 10.1016/j.stem.2016.01.017 26923202

[B212] Zhu Y. Y. Bian Y. Y. Gu W. J. Ni W. H. Wang C. Zhang C. N. (2020). The mir-184 level in the seminal plasma exosome of male infertility patients and its clinical significance. Zhonghua Nan Ke Xue 26 (8), 686–694. 33377728

